# Exploring the genetic variation of wheat-*Triticum timopheevii* introgression lines for flowering morphology traits for hybrid wheat use

**DOI:** 10.3389/fpls.2025.1621725

**Published:** 2025-08-19

**Authors:** Manel Othmeni, Surbhi Grewal, Jack Walker, Stella Hubbart-Edwards, Cai-yun Yang, Duncan Scholefield, Stephen Ashling, Ian P. King, Julie King

**Affiliations:** Nottingham Wheat Research Centre, School of Biosciences, University of Nottingham, Loughborough, United Kingdom

**Keywords:** wheat, hybrid wheat, *Triticum timopheevii*, introgression, flowering morphology, pollen size, genetic variation

## Abstract

**Introduction:**

The autogamous nature of wheat presents a significant challenge for hybrid wheat breeding, which relies on cross-pollination. To facilitate hybrid wheat production, it is essential to modify the floral morphology of wheat to promote outbreeding rather than inbreeding. While some genetic diversity for flower morphology exists within wheat, it is limited compared to the vast and largely untapped genetic variation found in its wild relatives for potentially all agronomically important traits, including flowering characteristics. The aim of this study was to identify genomic regions associated with flowering morphology traits in the wild relative *Triticum timopheevii*.

**Materials and methods:**

A set of 24 wheat-*T. timopheevii* introgression lines were screened for seven flowering-related traits: plant height, spike length, number of spikelets per spike, anther extrusion, filament length, anther length and pollen size.

**Results:**

A significant level of variation was observed among the population for all traits. Phenotyping highlighted the potential of *T. timopheevii* for enhancing filament length and pollen size for use in hybrid wheat production. Five introgression lines showed significant improvement in filament length or pollen size compared to the parental wheat lines. Through comparative analysis of introgression lines carrying different-sized segments of the same genome and linkage group, specific *T. timopheevii* genomic regions were identified as carriers of alleles responsible for increased filament length and smaller pollen grains. An inter-crossing strategy between two introgression lines, each carrying different-sized introgressions from Chromosome 5G of *T. timopheevii* with an overlapping region, was employed to generate a new introgression line with a smaller genomic segment believed to confer the desired trait. Phenotyping of plants with this smaller introgression confirmed the presence of an allele(s) responsible for producing smaller pollen grains.

**Discussion:**

This study demonstrates the potential of *T. timopheevii* to contribute valuable genetic variation for floral traits critical to hybrid wheat breeding, paving the way for improved outcrossing efficiency and enhanced hybrid seed production.

## 1 Introduction

In nature, wheat is simultaneously exposed to a combination of biotic and abiotic factors that challenge its yield potential. In a rapidly changing climate, there is a continuous need to develop resilient wheat varieties that can thrive under these pressures. Hybrid wheat varieties offer a promising solution, with the potential to significantly increase production levels through heterosis (hybrid vigor) (Singh et al., 2015; Longin et al., 2012; Ter Steeg et al., 2022). Moreover, these hybrids have demonstrated enhanced resilience to harsh climate conditions (Schneider et al., 2024). Hybrid wheat production requires inter-crossing of two distinct wheat genotypes. This process requires a pollen donor (male line) to be crossed with a male-sterile female line, where male sterility is achieved using chemical hybridizing agents, cytoplasmic male sterility, or genetic male sterility (Singh et al., 2015; Melonek et al., 2021).

Wheat is an autogamous species with an outcrossing probability of less than 1% (Curtis and Johnston, 1969; Pickett, 1993). This is primarily due to its cleistogamous flowering behavior where pollen is shed from the mature anther before the floret opens (De Vries, 1971; Curtis and Johnston, 1969). This characteristic poses a major challenge to developing a successful hybrid wheat system. To enable the transition from inbreeding to outbreeding, it is crucial to identify the genetic variability for flowering for future hybrid wheat production (Selva et al., 2020). For effective cross-pollination, the male and female plants must exhibit synchronized flowering times with open florets during anthesis (Murai et al., 2002).

Achieving a high outcrossing rate requires specific floral morphology traits (Langer et al., 2014; Garst, 2017; Boeven et al., 2018). Male plants should be taller than female plants, displaying high anther extrusion with long anthers containing abundant, highly viable pollen. Female plants should be shorter and exhibit gaping florets to maximize pollen capture, along with long, extruding stigmas characterized by extended receptivity (Whitford et al., 2013; Khare et al., 2018). Developing male and female ideotypes with these ideal floral traits necessitates considerable pre-breeding work and the identification of germplasm with the required genetic variation. However, since its domestication, wheat has experienced a significant reduction in genetic diversity compared to its wild relatives. In addition to breeding for increased yield and improved resistance and tolerance to biotic and abiotic stresses, breeders have historically selected varieties with cleistogamous flowers to prevent kernel shattering, a trait associated with open flowers (Vogel, 1941; Zhang et al., 2009). Consequently, genetic variation for the flowering morphology required for hybrid wheat breeding is quite limited within the existing wheat gene pool.

In contrast to domesticated wheat, wild relatives of wheat not only offer genetic variation for flowering traits but also serve as valuable sources of new alleles that can enhance heterosis in hybrid wheat breeding. There is already evidence demonstrating the genetic potential of wild relatives to improve flowering traits. For instance, the addition of chromosome 4R of rye (*Secale cereale*) has resulted in a 16% increase in anther size and a 33% improvement in pollen grain number (Nguyen et al., 2015).

One particularly promising wild relative is the tetraploid species *Triticum timopheevii* (2n=28, A^t^A^t^GG). *T. timopheevii* has shown its importance in enhancing several agronomic traits such as disease resistance. Resistance genes against leaf rust (Brown-Guedira et al., 2003; Leonova et al., 2010; Uhrin et al., 2012; Singh et al., 2017), stem rust (McIntosh and Gyarfas, 1971; Dyck, 1992; Khlebova and Barysheva, 2016), powdery mildew (Järve et al., 2000; Perugini et al., 2008) and Fusarium head blight (Steed et al., 2022) have been identified in *T. timopheevii* and successfully used in wheat improvement programs. In hybrid wheat breeding, *T. timopheevii* has mainly been used as an effective source of cytoplasmic male sterility (CMS) for female lines (Wilson and Ross, 1962) and is considered the best and most widely used CMS system available (Gupta et al., 2019; Suresh and Bishnoi, 2020; Melonek et al., 2021). However, until now, this species has not been extensively explored for its variation in flowering morphology traits.

At the Nottingham Wheat Research Centre (WRC) hundreds of wheat-wild relative introgression lines of several wild relative species have been developed (Grewal et al., 2018; 2019; 2020; 2021; King et al., 2017; 2019; 2022). These introgression lines contain one or more homozygous wild relative segments of varying sizes within hexaploid wheat backgrounds. The screening of these lines for hybrid wheat- related traits provides a unique opportunity to identify specific genomic regions of interest within the wild relative genome and facilitates their use in hybrid wheat breeding. In a recent study, King et al. (2022) published 99 genetically characterized wheat-*T. timopheevii* introgression lines using chromosome-specific Kompetitive Allele Specific PCR (KASP) markers. In the present study, the first 24 wheat-*T. timopheevii* introgression lines developed, each carrying different homozygous segments of *T. timopheevii*, were screened for flowering morphology traits with a focus on the traits required for male flowers in hybrid wheat production.

## 2 Materials and Methods

### 2.1 Plant material

Twenty-four homozygous wheat -*T. timopheevii* introgression lines, each carrying different-sized segments from the A^t^ and G genomes of *T. timopheevii* accession PI 94760 (United States National Plant Germplasm System), were selected for this study. These lines were previously characterized and released by the WRC (King et al., 2022). The annotation of the *T. timopheevii* introgressions present in the introgression lines followed the previously published data by King et al. (2022) (**Table 1**). However, new small introgressions identified via sequencing in some introgression lines during this work are also included and marked with an *in **Table 1**. The introgression lines were divided into three sub-populations according to the wheat parental lines present in their genetic background (**Table 1**). The three wheat parental genotypes Paragon, Chinese Spring and Highbury (obtained from the Germplasm Resource Unit, JIC, UK) were included as negative controls. Additionally, two wheat varieties, Apache and Piko, were selected as positive controls due to their known effectiveness as male pollinators in hybrid wheat breeding. All lines used in this experiment were spring wheat types, except for the two positive controls, Apache and Piko, which were winter wheat types. Phenotyping was carried out under glasshouse conditions with an average temperature of 25°C and 16-hour photoperiod.

**Table 1:** List of the three sub-populations of wheat-*T. timopheevii* homozygous introgression lines and description of the introgressions present.

Sub-population number	Wheat background genotypes	Introgression line name	Number of introgressions	Introgressions
Sub-pop 1	Paragon	Tim1	3	1A^t^*, 2A^t^.A5, 6A^t^.A9
		Tim3	3	2A^t^.A10, 5A^t^.A17, 7A^t^.A8
		Tim10	5	2A^t^.A10, 2G.B15, 3A^t^.A1, 5A^t^.A13, 5G.B14
		Tim15	2	1A^t^.A7, 1G.B1
		Tim18	2	5G.B15, 6G.B3
		Tim23	1	5A^t^.A10
		Tim24	2	3G.B1, 7G.B1
		Tim35	3	1A^t^.A9, 3G.B2, 7G.B3
Sub-pop 2	Paragon Chinese Spring	Tim4	4	5A^t^.A1, 5A^t^.A8, 6G.B4, 7A^t^.A1
		Tim7	1	7G.D2
		Tim8	1	5G.B3
		Tim9	2	5G.B3, 7A^t^.A9
		Tim12	2	2G.D5, 7G.D2
		Tim13	3	7A^t^.A1, 7A^t^.A12, 7G.D2
		Tim14	3	1A^t^.A5, 2A^t^.A1, 4G.B1
		Tim17	2	4G.B2, 5A^t^.A24
		Tim22	1	6A^t^.A7
		Tim26	1	2G.B12
		Tim28	2	1A^t^.A1, 5G.B8
		Tim33	2	2G.B14, 7A^t^.A5
Sub-pop 3	Paragon Highbury	Tim5	3	1A^t^.A12, 2G.D2, 3G.B4
		Tim6	2	2G.D4, 3G.B5
		Tim11	2	2G.D4, 6A^t^.A3
		Tim25	3	2G.D1, 5A^t^.A10, 6A^t^*

*New segment identified using skim-sequencing.

### 2.2 Plant phenotyping

Seven traits relevant to hybrid wheat breeding were evaluated in this study (**Table 2**).

**Table 2:** List of traits, methodology and experimental scale of each of the traits used in this study.

Trait	Methodology	Experiment scale
Plant height	Measured in centimeters (cm) on fully mature plants from the ground to the tip of the spike excluding awns.	Three plants per genotype (n=3)
Spike length	Total length of the spike excluding awns, measured in cm after complete heading.	Three main spikes of 3 plants per genotype (n=9)
Number of spikelets per spike	Number of spikelets per spike counted after plant heading.	Three main spikes of 3 plants per genotype (n=9)
Anther extrusion	Visually scored during flowering on a scale from 1 to 9 (1 – no anthers extruded; 9 = maximum anther extrusion).	Three main spikes of 3 plants per genotype (n=9)
Filament length	Visually scored during flowering on a scale of 1 to 9 (1 = no filament elongation; 9 = filament extrusion).	Three main spikes of 3 plants per genotype (n=9)
Anther length	Collected at flowering in a 3:1 solution of 95% ethanol: 100% acetic acid. Photographed using a stereomicroscope and length measured (μm) using ImageJ software.	Twelve anthers collected per plant of 3 plants per genotype (n=36)
Pollen size and viability	Three mature anthers were collected in 20 μl potassium iodide solution (0.2% KI: 1% 12 (w/v)). Pollen pictures were taken using a stereomicroscope at 20 times magnification.	Three anthers collected per plant of 3 plants per genotype. For each of the 3 plants per genotype, 10 random fields of view were photographed. Photos were analyzed using ImageJ software to determine pollen size (μm) and pollen viability (%) (n=30)

### 2.3 Plant genotyping

#### 2.3.1 KASP marker analysis

A set of 377 KASP markers (**Supplementary Table 1**) known to be polymorphic between wheat and *T. timopheevii* (King et al., 2022) were used to characterize the 24 wheat-*T. timopheevii* lines. DNA was extracted from 3–5 cm sections of 10-day old leaf material collected from each genotype into a 96-well collection plate. After being freeze-dried and ground using a TissueLyser (Qiagen), DNA extraction was performed as described in Thomson and Henry (1995). Genotyping was performed as described in Grewal et al. (2020). In summary, for each sample a 5 µl reaction containing: 1 ng genomic DNA, 2.5 µl KASP reaction mix (ROX), 0.068 µl primer mix and 2.43 µl nuclease free water was made using an automated PIPETMAX 268 (Gilson, United Kingdom). PCR was performed in a ProFlex PCR system (Applied Biosystems by Life Technology) and a QuantStudio 5 (Applied Biosystems) was used for fluorescence detection of the reactions. PCR conditions consisted of 15 min at 94°C; 10 touchdown cycles of 10 s at 94°C, 1 min at 65-57°C (dropping 0.8°C per cycle); then 35 cycles of 15 s at 94°C followed by 1 min at 57°C. The QuantStudio™ Design and Analysis Software V1.5.0 (Applied Biosystems) was used for data analysis. The raw genotyping data is available form the corresponding author on request. 

#### 2.3.2 Whole genome sequencing analysis

DNA extraction for sequencing was carried out using the extraction buffer (0.1 m Tris–HCl (pH 7.5), 0.05 m EDTA (pH 8.0), 1.25% SDS) of 10 days old leaf materials. Library preparation and DNA sequencing was performed by Novogene (UK) Company Limited. Each library was skim-sequenced to 0.05x coverage on a NovaSeq 6000 S4 flow cell with PE150 strategy. Sequence data analysis was carried out using two bioinformatics pipelines, to assess both the wheat background genome and the introgressed *T. timopheevii* genomic regions, as outlined by Coombes et al. (2023) and Adhikari et al. (2022), respectively. The former used IWGSC RefSeq v1.0 as the wheat reference genome assembly (IWGSC et al., 2018) to align the sequence reads whereas the latter pipeline used the recently published, chromosome-scale genome assembly of *T. timopheevii* (Grewal et al., 2024). This analysis allowed for the precise identification of the location and the size of the introgressed wild relative segments in the introgression lines. Raw skim-sequencing reads for T. timopheevii introgression lines have been deposited at the European Nucleotide Archive (ENA) under project accession PRJEB6515.

### 2.4 Statistical analysis

The experiment was performed using a randomized complete block design with a minimum of three replicates per genotype. The number of replicates varied according to the trait (**Table 2** - column experiment scale). Analysis of variance (one-way ANOVA) was used to examine differences between the mean values and computed at p-values < 0.05. Principal component analysis (PCA) and Pearson’s correlation coefficient analyses were used to determine the correlation level between all phenotyped traits. Statistical data analysis was performed using XLSTAT software (Lumivero, 2023).

## 3 Results

### 3.1 Genotyping analysis

**Figure 1:**
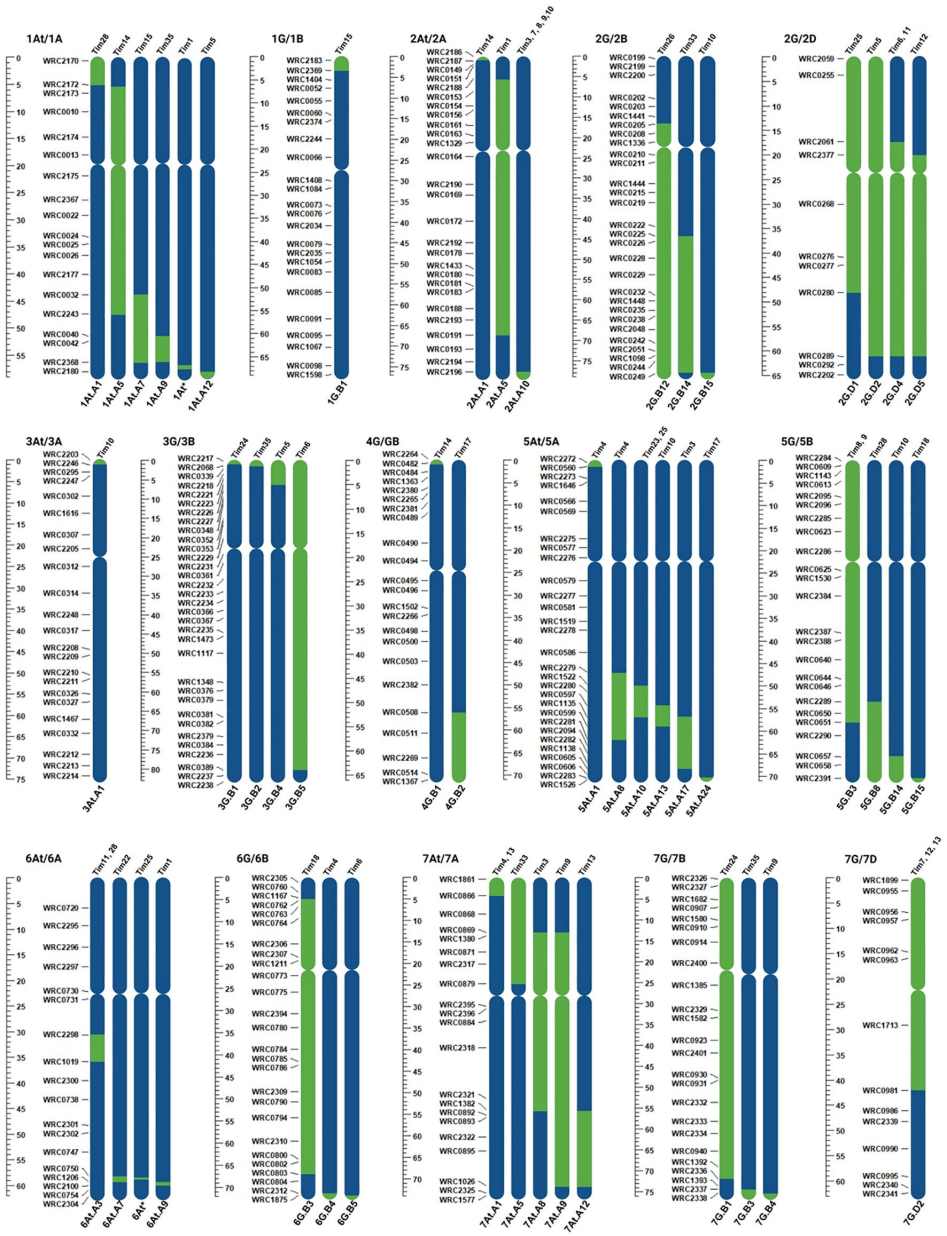
Schematic representation of introgressions from the *T. timopheevii* genome (green), showing the size and the location of the segments within the wheat genome (blue) of the 24 selected introgression lines.

KASP analysis of the 24 *T. timopheevii* introgression lines confirmed the presence of *T. timopheevii* segments (King et al., 2022), while skim-sequencing identified additional introgressions in two lines (**Figure 1**; **Supplementary Figure 1** **).** Small 1A^t^ and 6A^t^ segments were introgressed on the long arms of wheat chromosomes 1A and 6A in introgression lines Tim1 and Tim25, respectively (**Supplementary Figure 1**). These segments are small and located in regions not covered by the KASP markers used in the original study. Additionally, the 6At.A3 and 6At.A4 previously identified by KASP genotyping in Tim12 and Tim18, respectively (King et al., 2022) were not detected through sequencing, suggesting they were likely present in a heterozygous state and lost during plant multiplication.

In total, the 24 lines contained 50 unique segments, with introgressions from all *T. timopheevii* chromosomes except chromosome 4A^t^. Four lines carried a single introgression from chromosomes 3A^t^, 1G, 4G and 7G of *T. timopheevii*. The remaining 20 lines carried multiple introgressions from the remaining 10 chromosomes with several introgressions from the same chromosome showing overlap (**Figure 1**). All introgressed segments from the A^t^ genome chromosomes of *T. timopheevii* recombined with the A genome chromosomes of wheat. While the G genome chromosomes primarily recombined with the B genome of wheat, four introgressions occurred between the G and D genomes. Specifically, three introgressions were observed between 2D and 2G and one between 7D and 7G (**Figure 1**).

### 3.2 Variation and correlation analysis

**Figure 2:**
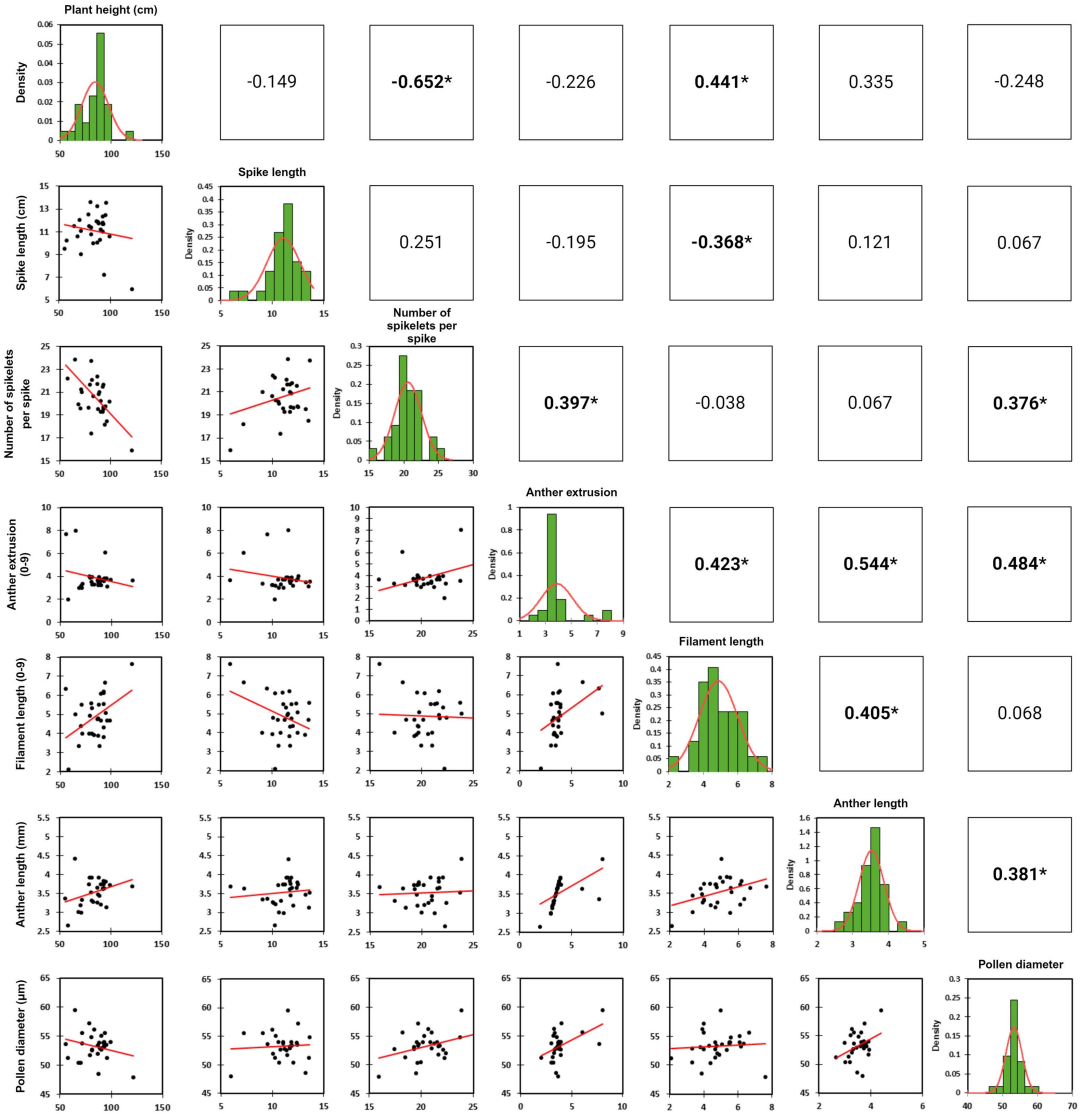
Scatter plots, trait distribution histograms and trait correlation coefficient of the phenotyped traits.

**Figure 3:**
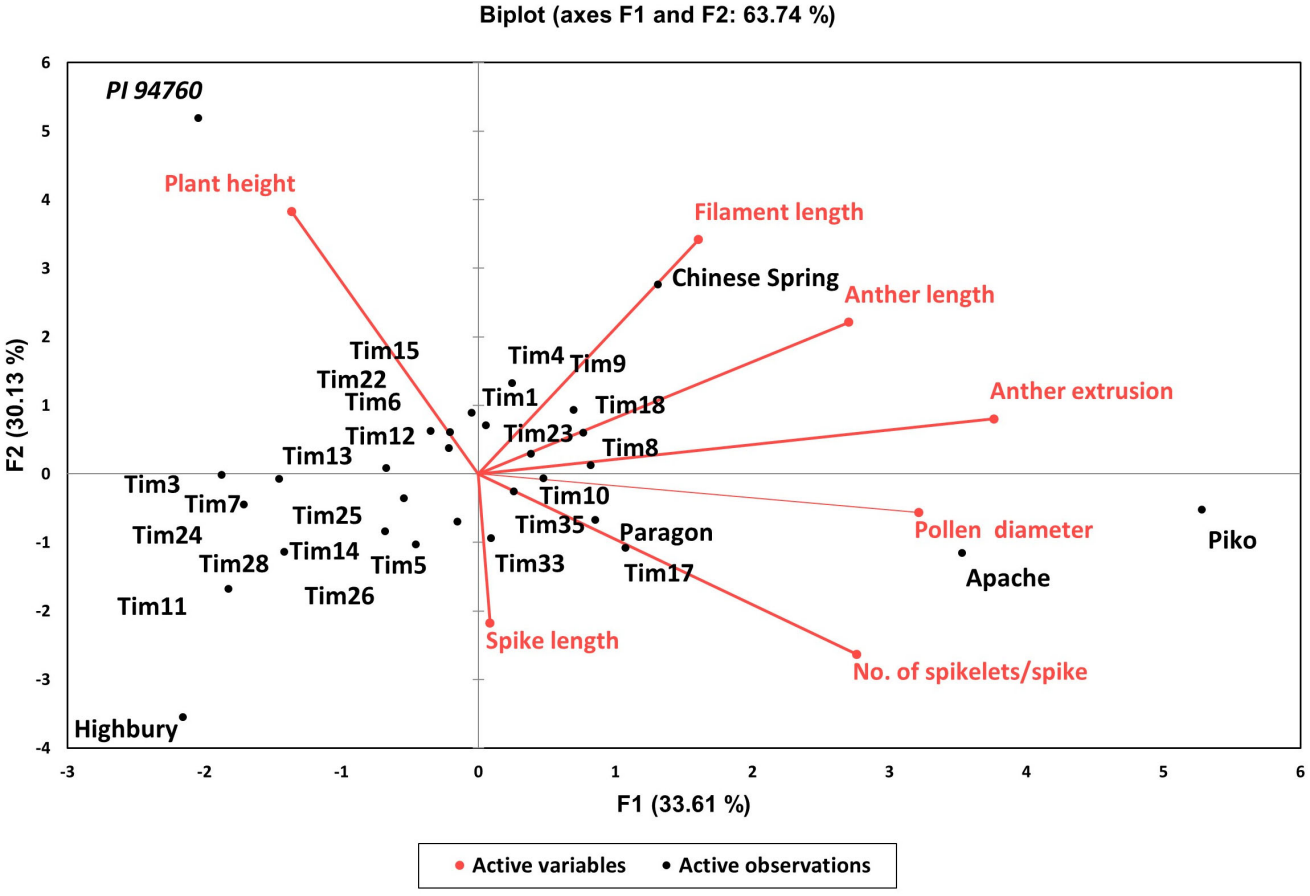
Two-dimensional principal component analysis plot for the wheat-*T. timopheevii* introgression lines.

The analysis of variance for the phenotyped *T. timopheevii* introgression lines showed highly significant variation across all traits (p <0.0001) (**Table 3**) with a normal distribution for all seven traits (**Figure 2**). To assess population structure, a Principal Component Analysis (PCA) was conducted using phenotypic data from the seven traits. The PCA explained 63.74% of the total observed variation in the wheat-*T. timopheevii* population (**Figure 3**). The PCA results demonstrated clear discrimination between the wild relative accession PI 94760 and the introgression lines, which clustered together closely with their parental wheat lines, primarily Paragon, present in the background of all lines. In contrast, the positive controls for male traits, Piko and Apache, formed a distinct cluster, showing a positive impact in anther extrusion, anther length, and pollen size traits according to the F1 axis, which explains 33.61% of the variation. The *T. timopheevii* accession PI 94760 exhibited the highest impact on plant height and filament length traits along the F2 axis, which accounts for 30.13% of the observed variation.

**Table 3:** Analysis of variance of the phenotyped traits.

Traits	Plant height	Spike length	No. of spikelets per spike	Anther extrusion	Filament length	Anther length	Pollen size
R^2^	0.850	0.844	0.765	0.957	0.594	0.800	0.647
F	13.501	12.832	7.737	90.944	3.486	9.400	4.359
Pr >F	<0.0001	<0.0001	<0.0001	<0.0001	<0.0001	<0.0001	<0.0001

Pearson’s correlation analysis of all phenotyped traits revealed absolute correlation values ranging from 0.05 to 0.65 (**Figure 2**). The strongest significant positive correlation was observed between anther extrusion and anther length (0.544), while the strongest significant negative correlation occurred between the number of spikelets per spike and plant height (-0.652). Additionally, anther extrusion showed positive correlations with filament length, pollen size and number of spikelets per spike. Pollen size was also positively correlated with the number of spikelets per spike and anther length (**Figure 2**).

### 3.3 Single trait analysis

**Figure 4:**
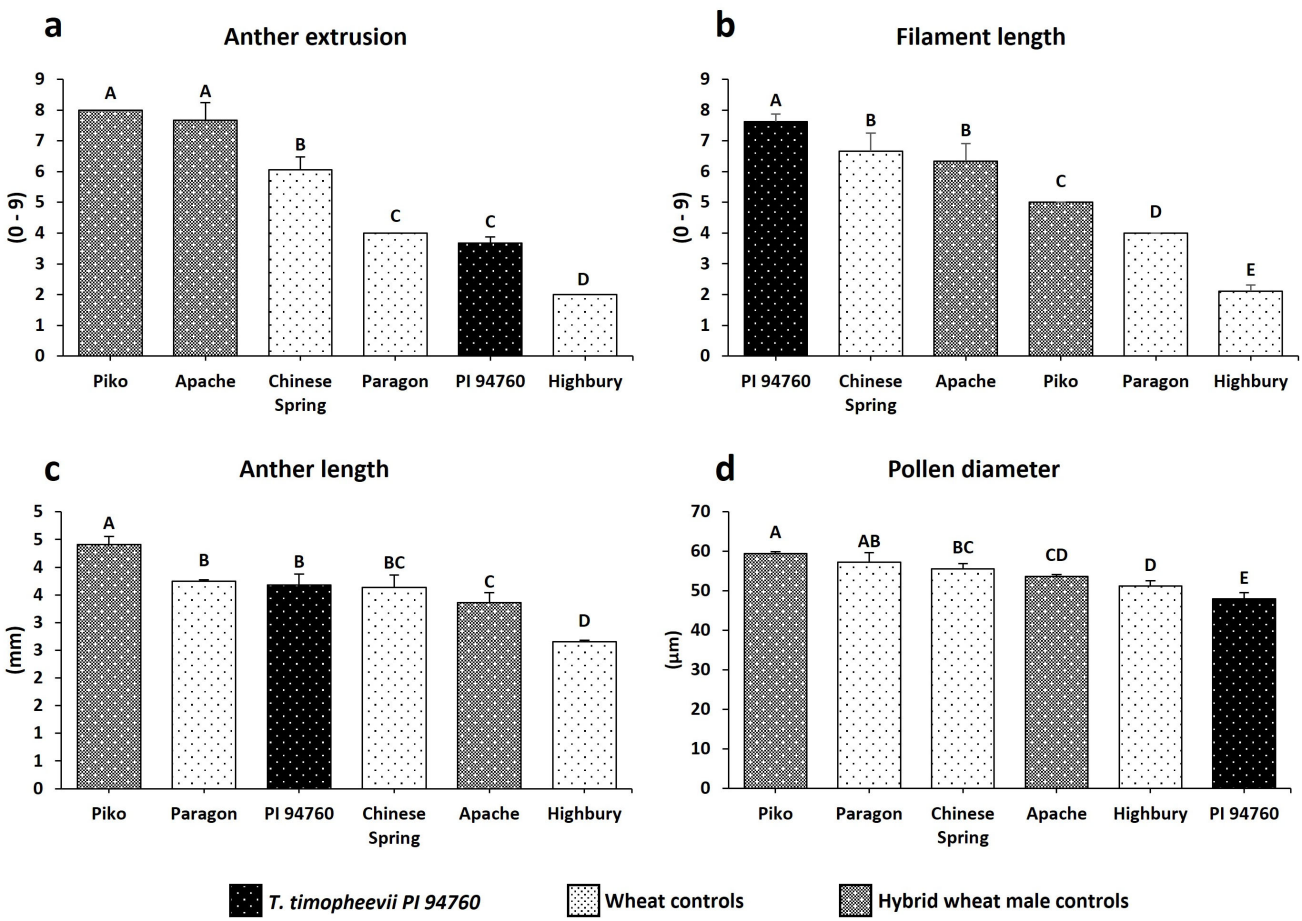
Histogram plots of the traits evaluated for *T. timopheevii* and the wheat controls. **(a)** Anther extrusion **(b)** Filament length **(c)** Anther length **(d)** Pollen diameter. Letters above the bars indicate groupings based on statistical significance. Bars that share the same letter are not statistically different from each other. Bars with different letters are significantly different at p<0.05.

The *T. timopheevii accession* PI 94760 (originally used to generate the introgression lines), showed a significant increase in filament length and smaller pollen grains compared to all wheat controls (**Figures 4b, c**). No significant impact on either anther extrusion or anther length traits was observed from the wild species (**Figures 4a, d**). To determine the effect of *T. timopheevii* introgressions, particularly on the two traits identified as of interest, filament length and pollen size, lines were divided into three sub-populations based on the three different wheat parental backgrounds (**Table 1**) and analyzed separately. Five introgression lines showed either increased filament length or reduced pollen grain size compared to the parental lines, as summarized in **Table 4**.

**Table 4:** Summary list of *T. timopheevii* introgression lines of interest for filament length and pollen size traits.

Trait	*T. timopheevii* introgression lines	Introgression
Filament length	Tim6	2G.D4, 3G.B5
	Tim18	5G.B15, 6G.B3
	Tim23	5A^t^.A10
Pollen size	Tim24	3G.B1, 7G.B1
	Tim28	1A^t^.A1, 5G.B8

#### 3.3.1 Filament length

**Figure 5:**
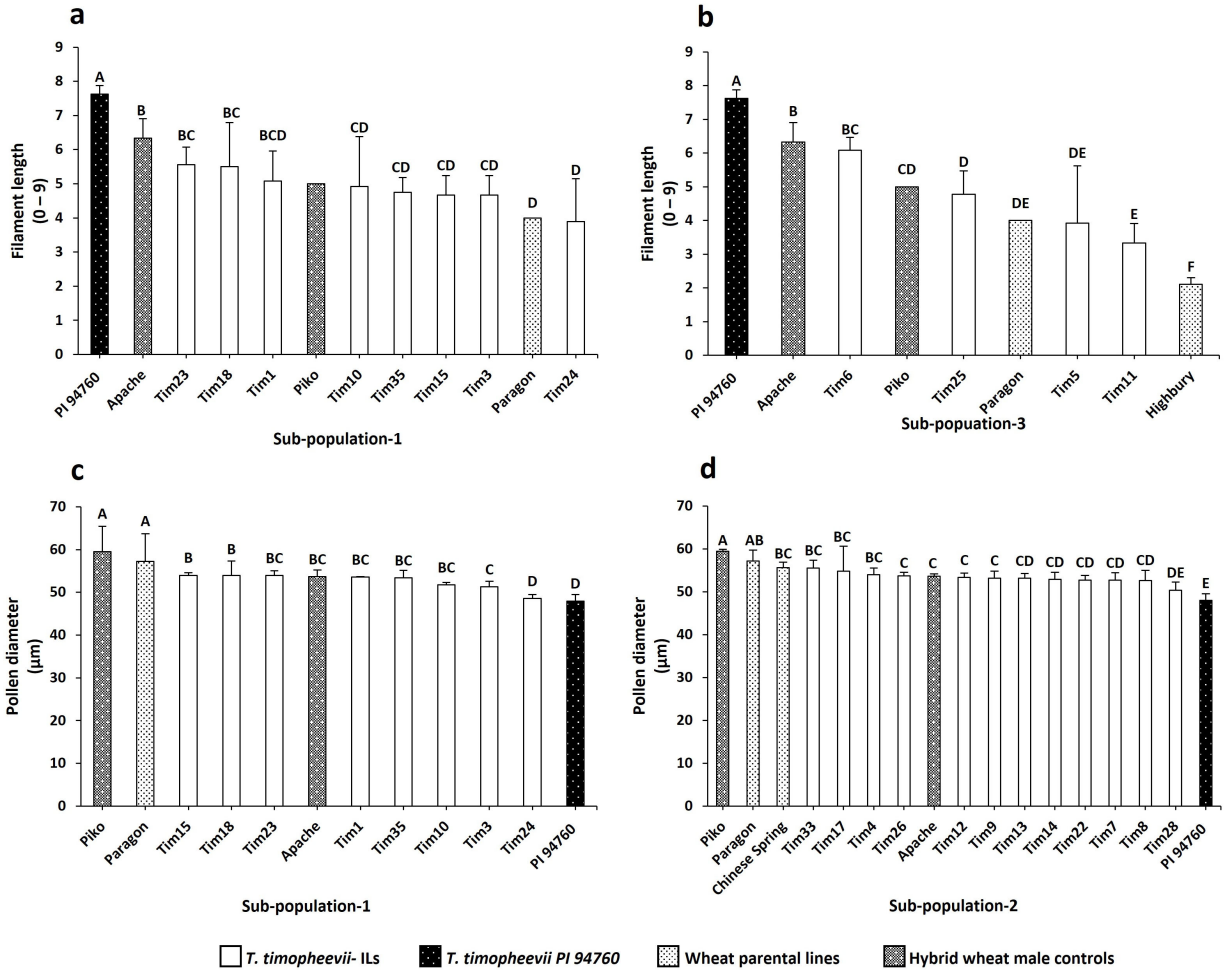
Histogram plots of the traits of interest within the screened sub-populations. **(a)** Filament length in Sub-population 1 **(b)** Filament length in Sub-population 3 **(c)** Pollen diameter in Sub-population 1 **(d)** Pollen diameter in Sub-population 3. Letters above the bars indicate groupings based on statistical significance. Bars that share the same letter are not statistically different from each other. Bars with different letters are significantly different at p<0.05.

In Sub-population 1, Tim23 and Tim18 exhibited a significantly longer filament compared to the parental wheat line Paragon (**Figure 5a**), showing an increase in length of 22%. In Sub-population 3, which included only four lines, Tim6 demonstrated a significantly longer filament than both parental genotypes, Paragon and Highbury, as well as the positive control Piko, with an improvement of up to 52% (**Figure 5b**).

#### 3.3.2 Pollen size

In Sub-population 1, Tim24 had significantly smaller pollen grains compared to the parental line Paragon with a 14% reduction in pollen size (**Figure 5c**). In addition, phenotyping of Sub-population 2 showed that Tim28 had significantly smaller pollen grains than both Paragon and Chinese Spring, as well as the two positive controls Apache and Piko (**Figure 5d**). A reduction of 9% and 6% in pollen diameter was observed when compared to the Chinese Spring and Apache controls, respectively. Within the same sub-population, Tim8, while not significantly smaller, still showed smaller pollen grains than all the wheat controls included in the screening (**Figure 5d**). Both Tim8 and Tim28 contained introgressions from *T. timopheevii* chr5G. Sequencing and KASP genotyping showed the presence of an overlapping region from the long arm of *T. timopheevii* chromosome (Chr) 5G between these two lines (**Figure 1**; 5G.B3 and 5G.B8).

Two additional lines screened in Sub-population 1, Tim10 and Tim18, and one line in Sub-population 2, Tim9, also contained segments from the long arm of *T. timopheevii* Chr5G (**Figure 1**, 5G/5B). Although Tim9 carried the same 5G introgression (5G.B3) as Tim8, its pollen size was larger than Tim8 but still smaller than the wheat parental lines Paragon and Chinese Spring (**Figure 5d**). Neither Tim10 nor Tim18 from Sub-population 1 showed a small pollen grain phenotype, as their introgressions did not include the overlapping region found in Tim8 and Tim28. Thus, this indicates that the overlapping region within *T. timopheevii* Chr5G introgression potentially carries a gene(s) responsible for the smaller pollen grain phenotype.

### 3.4 The identification of the region of interest responsible for the smaller pollen size trait in *T. timopheevii*

**Figure 6:**
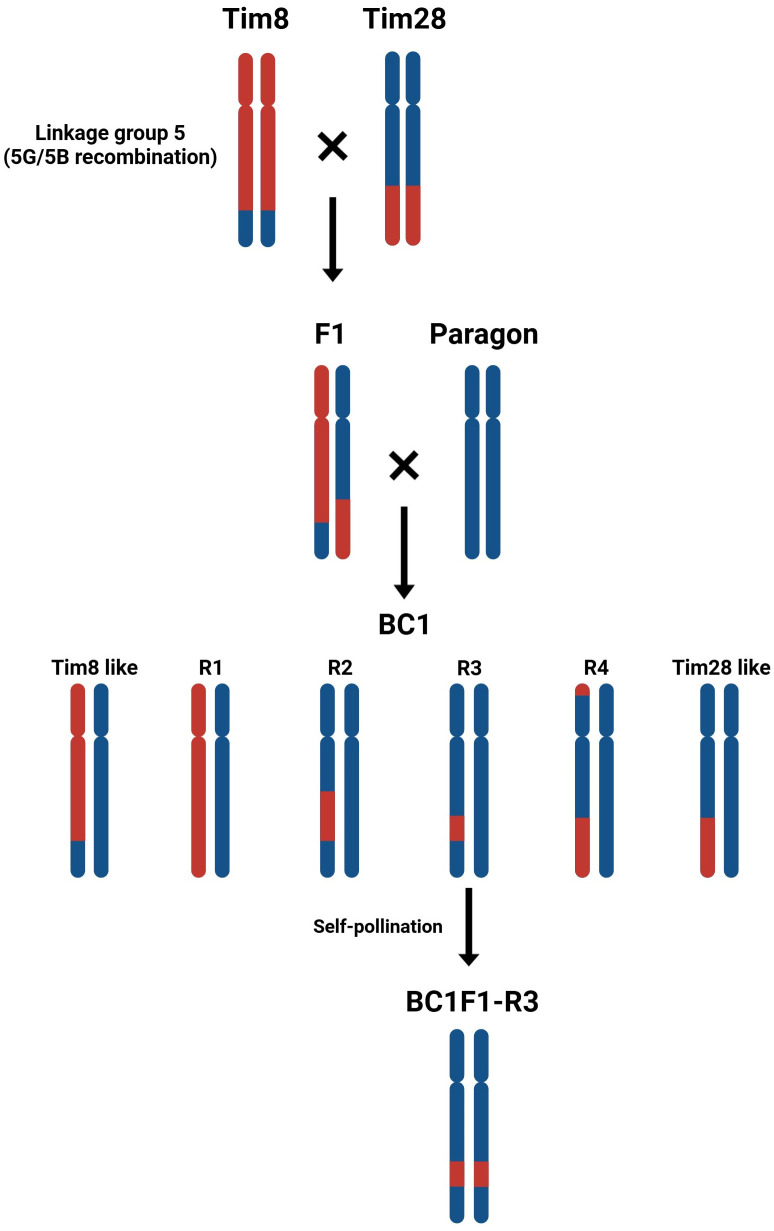
Inter-crossing diagram of Tim8 and Tim 28. The chromosomes 5B of wheat and 5G of *T. timopheevii* are represented in blue and red, respectively.

In order to reduce the size of the introgression carrying the gene(s) responsible for reduced pollen grain size, introgression lines Tim8 and Tim28 were inter-crossed (**Figure 6**) generating 139 F_1_ seed heterozygous for both introgressions. Eight F_1_ seed were germinated and crossed to the recurrent parent Paragon, generating 166 BC_1_ seed. Eighty-seven BC_1_ plants were genotyped with 20 KASP markers specific to *T. timopheevii* Chr5G. Genotyping showed that 81 of the 87 BC_1_ plants (93%) contained a heterozygous introgression from Chr5G. Of these, 35 plants (40%) contained the same introgression present in Tim8, and 19 plants (22%) contained the same introgression as Tim28. Notably, 27 plants (31%) were found to contain new introgressions derived from recombination between the *T. timopheevii* chromosomes segments in Tim8 and Tim28. These new introgressions were characterized into 4 distinct groups (R1 to R4: **Figure 6**). The new segments in R1 and R3 were generated by recombination within the overlapping region between the two different *T. timopheevii* Chr5G introgressions. In contrast, the shortened segments in R2 and R4 resulted from recombination between the *T. timopheevii* Chr5G introgression in Tim8 and the wheat 5B region in the Tim28, rather than through recombination between the two *T. timopheevii* segments.

**Figure 7:**
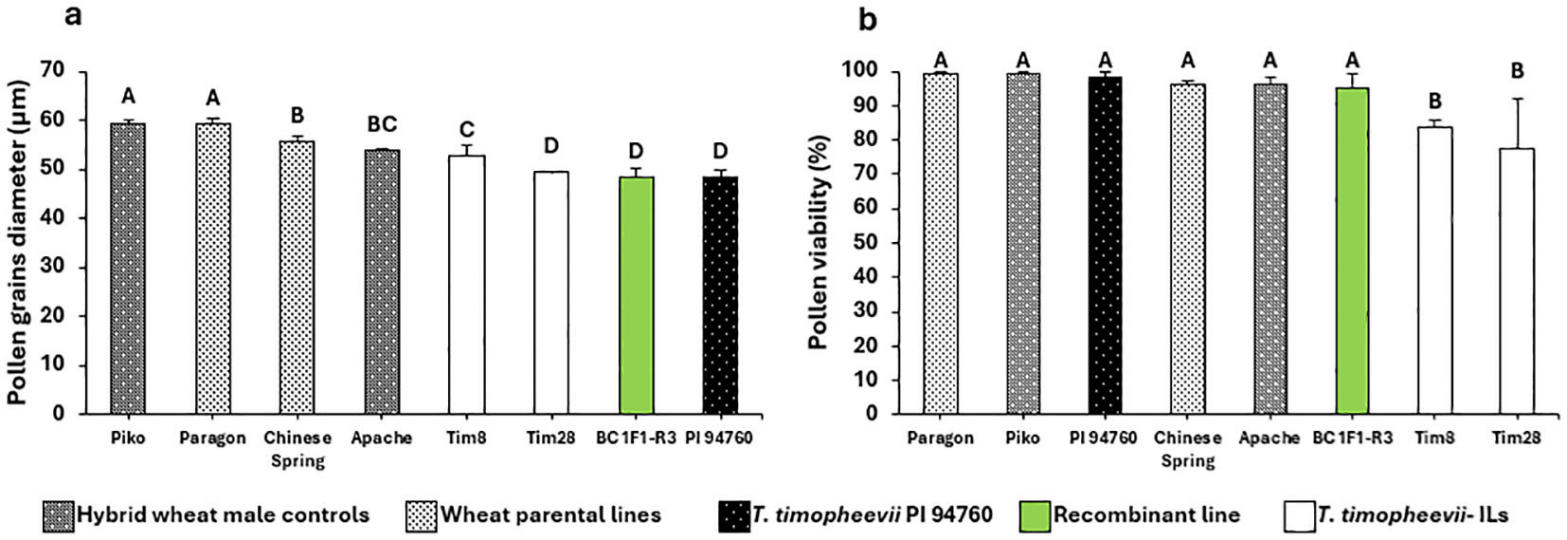
Histogram plots for **(a)** pollen grain size and **(b)** pollen grain viability. Letters above the bars indicate groupings based on statistical significance. Bars that share the same letter are not statistically different from each other. Bars with different letters are significantly different at p<0.05.

Four of the R2 BC_1_ plants, carrying a significantly reduced *T. timopheevii* introgression derived from the overlapping region between Tim8 and Tim28, were self-fertilized to generate BC_1_F_1_ progeny. Twenty BC_1_F_1_ plants were then genotyped with three KASP markers, WRC2289, WRC0650 and WRC0651, which map within the overlap at physical positions 53.4 and 58.0 Mbp. Genotyping identified three lines with a homozygous shortened introgression. When these three lines, along with the parental lines and wheat controls, were screened for pollen size, all the introgression lines were found to have the smaller pollen grain phenotype (**Figure 7a**). The pollen viability was also calculated for the lines showing the smaller pollen phenotype and compared to the viability of the wheat parents and controls (**Figure 7b**). The viability of the pollen produced by both Tim8 and Tim28 was found to be lower than that of all wheat controls. However, the pollen viability of plants with the shortened introgression was at a comparable level to the wheat controls (**Figure 7b**).

## 4 Discussion

### 4.1 Genetic variation and trait association

The identification of lines with favorable male floral traits remains a bottleneck for hybrid wheat breeding (Boeven et al., 2016), where a high level of outcrossing is required. Here, we studied 24 wheat-*T. timopheevii* introgression lines for seven traits known to influence pollination, to assess the potential genetic variation for floral morphology contributed by this wild relative species (**Tables 1**, **2**). Unlike previous studies that primarily focused on floral traits within wheat populations (Langer et al., 2014; Tadlock, 2015; Denisow et al., 2022; Sade et al., 2022; Garst et al., 2023), this study examined the variation present within a set of *T. timopheevii* introgression lines alongside several wheat controls. The results highlight the importance of considering population structure when assessing genetic variation. A highly significant level of genotypic variation was identified for all seven phenotyped traits. This genotypic effect could be attributed to the presence of the various introgressions from the A^t^ and/or the G genome of *T. timopheevii* within these lines. Similarly, a high level of variation for plant height, number of spikelets per spike, anther extrusion and filament length has also been reported in a population of elite wheat germplasm (Langer et al., 2014).

Trait correlations indicated that caution is needed when introducing a specific trait, as it may adversely affect other desirable traits. For example, plant height has been shown to influence male cross-ability in hybrid wheat. Dwarfing or reduced height (*Rht*) genes have a documented negative effect on anther extrusion (Beri and Anand, 1971; Langer et al., 2014; Boeven et al., 2016). Lu et al. (2013) further demonstrated that a quantitative trait locus (QTL) for low anther extrusion co-localized with the dwarfing allele *Rht-B1*. In this study, although plant height positively correlated with filament length, no significant correlation with anther extrusion was observed. Such correlations must be considered when selecting plants for anther extrusion, as anther extrusion and filament length are known to be positively and tightly linked traits, as documented in several studies (Beri and Anand, 1971; Langer et al., 2014; Boeven et al., 2018; Denisow et al., 2022) and corroborated by our results (**Figure 2**). While these traits may not be genetically linked, they often correlate due to their biological role in the pollination process. Additionally, when selecting plants with smaller pollen size, a desirable trait for improved pollen dispersal during pollination, care must be taken not to compromise the anther extrusion trait. Our findings revealed a significant positive correlation between pollen size and anther extrusion, underscoring the need for a balanced selection strategy.

### 4.2 Association between phenotyping and genotyping analysis

KASP genotyping of the 24 introgression lines showed that while they did not cover the entire genome of *T. timopheevii*, they included a range of differently sized segments from both the A^t^ and G genomes. Whole genome sequencing of the introgression lines identified two additional small segments in Tim1 and Tim25, originating from Chr1A^t^ and Chr6A^t^, respectively. These segments were between 10–20 Mbp in size and located in regions not covered by the KASP markers used in the original characterization of these lines (King et al., 2022). Conversely, two segments previously identified in Tim12 and Tim18 from Chr6A^t^ (6At.A3 and 6At.A4, respectively) were not detected by sequencing analysis. This discrepancy suggests that these segments were initially present in a heterozygous state and potentially lost during plant multiplication, which aimed to bulk the introgression lines seed. This finding underscores the importance of sequencing analysis in further confirming and characterizing the introgression lines.

For individual trait analysis, the three sub-populations, based on the wheat parental genotypes, were analyzed separately (**Table 1**). Five lines showed improvements in the two traits of interest associated with *T. timopheevii* PI 94760 accession, i.e., filament length and pollen size (summarized in **Table 4**). Both traits are of key importance for hybrid wheat production. Genotyping and phenotyping data were then integrated to assess whether specific genomic regions could be linked to these traits of interest.

#### 4.2.1 Filament length

Three plants exhibited longer filament length; Tim18 and Tim23 from Sub-population 1 and Tim6 from Sub-population 3 (**Table 4**; **Figures 5a, b**). Tim6 contains two introgressions (from Chr2G and Chr3G). Only the Chr3G introgression (**Figure 1**; 3G.B5) is partially unique to Tim6 suggesting that the gene(s) conferring a 52% increase in filament length may be located within this introgression.

Tim18 contains two introgressions from Chr5G and Chr6G, neither of which can be completely ruled out. Equivalent Chr5G introgressions are present in two other lines, Tim10 and Tim28, both of which exhibited shorter filament length. Thus, it is unlikely that the Chr5G introgression in Tim18 is carrying a gene(s) for longer filament length. However, Tim18 also carries a large and unique introgression from Chr6G which could potentially harbor gene(s) contributing to this phenotype. Further work is needed to reduce the size of this large Chr6G introgression and create lines with smaller introgressions covering different regions of Chr6G, ideally with overlapping segments. Phenotyping these lines would help identify which genomic regions are important for the increase in filament length.

Tim23 possesses an Chr5A^t^ introgression that is also present in several other lines screened for filament length, including Tim4 and Tim25. KASP genotyping indicates that the Chr5A^t^ introgressions in Tim4 and Tim25 completely match the introgression in Tim23 (**Figure 1**). Although neither of these two lines showed a significantly longer filament, both exhibited longer filaments than those of the wheat lines in their background (Paragon, Chinese Spring and Highbury). Thus, the Chr5A^t^ introgression requires further investigation to determine if it carries genetic diversity for filament length. Crossing all three introgression lines (Tim23, Tim4 and Tim25) several times to Paragon to achieve a more uniform genetic background, would be an essential first step in this investigation.

Unlike anther extrusion, which is known to be a polygenic trait (Lu et al., 2013; Skinnes et al., 2010), the genetic basis of filament length in wheat has not been extensively studied. In *Arabidopsis thaliana*, genetic control of filament length is mainly regulated by hormones that influence gene expression and cell elongation within the filament (Ge et al., 2010). Previous studies have consistently highlighted the significant positive correlation between anther extrusion and filament length (Beri and Anand, 1971; Langer et al., 2014; Boeven et al., 2018; Denisow et al., 2022), a finding also supported by this study. This correlation is not surprising, as anther extrusion is a combined effect of flower opening and filament elongation, which pushes the anthers out of the floret during anthesis (Joppa et al., 1968; Beri and Anand, 1971; De Vries, 1974; Sage and Isturiz, 1974; Boeven et al., 2016). In wheat, flower opening during anthesis is driven by lodicule swelling (De Vries, 1971). While both lodicule enlargement and filament elongation are triggered by water influx associated with potassium accumulation, this osmotic influx could be independently regulated in these organs (Heslop-Harrison and Heslop-Harrison, 1996). Interestingly, despite the increased filament length in Tim6, Tim18 and Tim23, this did not translate to better anther extrusion in these introgression lines, unlike the wild relative species *T. timopheevii*. This suggests that flower opening, filament length and anther extrusion could be under separate genetic control mechanisms.

#### 4.2.2 Pollen size

Hexaploid wheat typically produces relatively large pollen grains, with a diameter of approximately 58 µm (Goss, 1968; De Vries, 1971). In this study, the wheat controls had an average pollen diameter of 55 µm, ranging from 51 µm in Highbury to 59 µm in diameter for Piko, with Paragon, the common wheat parent of all the introgression lines, having a pollen diameter of 57 µm. While the weather conditions significantly influence pollen dispersal (Faegri and Van Der Pijl, 1979), pollen size also plays a crucial role, directly affecting on the aerodynamic properties of the pollen grain and consequently, pollination efficiency (D’Souza, 1970; De Vries, 1971; Waines and Hegde, 2003). Size differences impact the fall rate of pollen and therefore, the distance it can travel from the source. The relatively heavy pollen grains of hexaploid wheat (De Vries, 1971) have a sink rate of 55–60 cm/sec over 1 meter in still air (Lelley, 1966). As a result, more than 90% of pollen grains typically settle within 3 meters of the source (Hegde and Waines, 2004). Thus, in a hybrid wheat system, smaller pollen grains are desirable as they can travel further from the source, promoting optimum dispersal to the female plants and enhancing the potential of cross-pollination.

Two introgression lines, Tim24 (Sub-population 1) and Tim28 (Sub-population 2) showed a significantly smaller pollen grain phenotype compared to the wheat parental lines, showing a phenotype more similar to the *T. timopheevii* accession (**Figures 5c, d**). Comparing the genomic segments present in these lines with those not exhibiting the smaller pollen phenotype suggests that several regions of the *T. timopheevii* genome could be of interest for this trait. Tim24 carries two introgressions from Chr3G and Chr7G. The Chr3G introgression (3G.B1) is also present in Tim5, Tim6 and Tim35, none of which showed the small pollen grain phenotype. Hence, this introgression can be excluded as a candidate for this trait. The second introgression in Tim24 consists of a large Chr7G segment (7G.B1) that is partially unique to this line (**Figure 1**). Although Tim7, Tim12 and Tim13 also contain a large Chr7G introgression (7G.D2), their 7G segments recombined with the D genome, whereas the segment in Tim24 recombined with the B genome (**Figure 1**). There is a notable overlap between the 7G.B1 and 7G.D2 segments. Since the Chr7G introgression in Tim24 is larger than those in Tim7, Tim12 and Tim13 introgression lines, it was possible to narrow down the region of interest on the long arm of 7G chromosome of *T. timopheevii*. Sequencing data estimates the size of this region at approximately 165 Mbp. While the two Chr7G segments (7G.B1 and 7G.D2) recombined with different wheat genomes (B and D, respectively), the large size of these introgressions provides an opportunity to narrow the 7G region in Tim24 through targeted crossing. This strategy may help identify the specific gene(s) responsible for the smaller pollen grain phenotype.

Tim28 carries two introgressions from Chr1A^t^ and Chr5G, both of which are potential candidates for contributing to the smaller pollen grain phenotype. The Chr1A^t^ segment (1A^t^.A1) is a small introgression of approximately 30 Mbp that has recombined with the short arm of wheat chromosome 1A and is unique to Tim28. Additionally, four introgression lines (Tim8, Tim9, Tim10 and Tim18), contain introgressions from Chr5G, but only introgression 5G.B3 overlaps with the introgression in Tim28 (5G.B8). Although Tim8 did not exhibit significantly smaller pollen grains compared to the wheat controls, it had the next smallest pollen size in Sub-population 2 after Tim28. Through an inter-crossing program between Tim28 and Tim8, a recombinant introgression line (BC_1_F_1_-R3) was developed, carrying the genomic region of interest (**Figure 6**). When homozygous, phenotyping of BC_1_F_1_-R3 showed that it retained the small pollen grain phenotype, providing strong evidence that the reduced Chr5G region contains a gene(s) associated with smaller pollen size. Interestingly, the recombinant line BC_1_F_1_-R3 showed a small pollen grain size more similar to Tim28 than Tim8 (**Figure 7**). Since Tim28 produced smaller pollen grains than Tim8, it is possible that Tim8 carries a second gene conferring a larger pollen size outside the region of overlap with Tim28. In the recombinant lines with the smaller introgressions, this gene will no longer be present. The viability of pollen in a hybrid wheat system is also of significant importance as a reduction in viability will lead to a decrease in pollination efficiency (Whitford et al., 2013). Tim8 and Tim28 both showed reduced viability compared to the wheat parents and controls. However, pollen viability of the lines carrying the reduced introgression was higher than in either Tim8 or Tim28 and was equivalent to the viability seen in the wheat controls. Thus, the larger Chr5G introgressions in Tim8 and Tim28 appear to have a detrimental effect on pollen viability either by introducing a gene(s) contributing to this negative effect or by replacing a gene(s) from wheat itself. Except for Tim33, this negative effect was limited to the lines carrying the larger Chr5G introgressions (**Supplementary Figure 2**).

It is generally assumed that once a wild relative chromosome segment has been introgressed into wheat, reducing the size of the segment is not feasible due to the lack of recombination between the wheat and the wild relative genome. However, a strategy of inter-crossing two lines with overlapping introgressions was used by Sears (1956) to reduce the size of an *Aegilops umbellulata* segment carrying rust resistance. This approach has also been used to reduce the size of introgressions from rye (Koebner et al., 1986; Koebner and Shepherd, 1988; Anugrahwati et al., 2008), *Haynaldia villosa* (Lucaszewski and Cowger, 2017) and *Aegilops sharonensis* (Khazan et al., 2020). In this study, the presence of an overlapping region between the two wild relative segments in Tim8 and Tim28 enabled recombination to occur between *T. timopheevii* introgressions, i.e., like with like. Interestingly, while shortened introgressions were obtained through recombination between the two overlapping *T. timopheevii* Chr5G segments, recombination also occurred between *T. timopheevii* Chr5G and wheat chromosome 5B. As expected, recombination frequency was higher between the two *T. timopheevii* segments (24 out of 87, 26%) compared to recombination between *T. timopheevii* and wheat (3 out of 87, 5%). Both the B genome of hexaploid wheat and the G genome of *T. timopheevii* are believed to have originated from an *Aegilops* sp*eltoides*-like progenitor (Ogihara and Tsunewaki, 1988; Dvořák et al., 1989). However, chromosome pairing studies suggested the G genome of *T. timopheevii* and the S genome of *Ae.* sp*eltoides* are more closely related to each other than to the wheat B genome (Rodriguez et al., 2000).

## 5 Conclusion

The phenotyping of a population of 24 introgression lines carrying different-sized segments of the A^t^ and G genomes of *T. timopheevii*, alongside the wild relative parental accession PI 94760, highlighted the potential of this accession for enhancing filament length and pollen size traits in hybrid wheat breeding. Several genomic regions of interest have been identified that may harbor alleles with these traits. In some cases, further work is required to separate introgressed segments into different plants to refine the location of the trait to a specific genomic region. This utilized the first 24 wheat-*T. timopheevii* introgression lines developed at the WRC. Since then, the number of available lines has increased to over 150, offering opportunities to investigate additional genomic regions of interest, including those not covered in this study. For example, some identified introgressions of interest are relatively large. Where smaller introgressions of these genomic regions are now available, screening them could help narrow down the region of interest. In cases where smaller introgressions are not yet available, further work will be necessary to reduce the size of these introgressions. The strategy used in this work to reduce the size of an introgression from Chr5G carrying variation for smaller pollen size could serve as a model approach. The availability of the fully annotated genome assembly of *T. timopheevii* accession PI 94760 (Grewal et al., 2024) will further facilitate gene identification. Further work may also focus on generating combinations of different introgressions, including those from other species. For example, combining an introgression from *T. timopheevii* that enhances filament length with an introgression from *Aegilops mutica* associated with floret gaping (in preparation) could be a promising strategy for hybrid wheat development.

## Data Availability

The datasets presented in this study can be found in online repositories. The names of the repository/repositories and accession number(s) can be found in the article/**Supplementary Material**.

## Supplementary material

Supplementary Figure 1 KASP marker identification of introgressions present in the 24 wheat-T*. timopheevii* introgression lines screened for flowering morphology.

Supplementary Figure 2 Percentage pollen viability for the wheat parents and controls and the wheat-*T. timopheevii* introgression lines.

## References

[B1] Adhikari L. Raupp J. Wu S. Wilson D. Evers B. Koo D.. (2022). Genetic characterization and curation of diploid A-genome wheat species. Plant Phys. 188, 2101–2114. doi: 10.1093/plphys/kiac006, PMID: 35134208 PMC8968256

[B2] Anugrahwati D. R. Shepherd K. W. Verlin D. C. Zhang P. Mirzaghaderi G. Walker E.. (2008). Isolation of wheat-rye 1RS recombinants that break the linkage between the stem rust resistance gene *SrR* and secalin. Genome 51, 341–349. doi: 10.1139/G08-019, PMID: 18438437

[B3] Beri S. M. Anand S. C. (1971). Factors affecting pollen shedding capacity in wheat. Euphytica 20, 327–332. doi: 10.1007/BF00056096

[B4] Boeven P. H. Longin C. F. H. Leiser W. L. Kollers S. Ebmeyer E. Wurschum T. (2016). Genetic architecture of male floral traits required for hybrid wheat breeding. Theor. Appl. Genet. 129, 2343–2357. doi: 10.1007/s00122-016-2771-6, PMID: 27553082

[B5] Boeven P. H. G. Würschum T. Rudloff J. Ebmeyer E. Longin C. F. H. (2018). Hybrid seed set in wheat is a complex trait but can be improved indirectly by selection for male floral traits. Euphytica 214, 110. doi: 10.1007/s10681-018-2188-2181

[B6] Brown-Guedira G. L. Singh S. Fritz A. K. (2003). Performance and mapping of leaf rust resistance transferred to wheat from *Triticum timopheevii* subsp *Armeniacum* . Phytopathol. 93, 784–789. doi: 10.1094/phyto.2003.93.7.784, PMID: 18943158

[B7] Coombes B. Fellers J. P. Grewal S. Rusholme- Pilcher R. Hubbart-Edwards S. Yang C. Y.. (2023). Whole-genome sequencing uncovers the structural and transcriptomic landscape of hexaploid wheat/*Ambylopyrum muticum* introgression lines. Plant Biotechnol. J. 21, 482–496. doi: 10.1111/pbi.13859, PMID: 35598169 PMC9946142

[B8] Curtis B. C. Johnston D. R. (1969). Hybrid wheat. Sci. Am. 220, 21–29. doi: 10.1038/scientificamerican0569-21 5777205

[B9] D’Souza L. (1970). Studies in the suitability of wheat as pollen donor for cross pollination compared with rye, Triticale and Secalotricum. Z. fur Pflanzenzuchtung 63, 246–269.

[B10] Denisow B. Masierowska M. Winiarczyk K. Rakoczy-Trojanowska M. (2022). The pollen dispersal ability for cross-pollination in winter wheat (*Triticum aestivum* L.) is related to anther extrusion capability rather than to pollen output. S. Afr. J. Bot. 148, 283–292. doi: 10.1016/j.sajb.2022.04.031

[B11] De Vries A. P. (1971). Flowering biology of wheat, particularly in view of hybrid seed production - A review. Euphytica 20, 152–170. doi: 10.1007/BF00056076

[B12] De Vries A. P. (1974). Some aspects of cross-pollination in wheat (*Triticum aestivum* L.). 3. Anther length and number of pollen grains per anther. Euphytica 23, 11–19. doi: 10.1007/BF00032735

[B13] Dvořák J. Zhang H.-B. Kota R. S. Lassner M. (1989). Organization and evolution of the 5S ribosomal RNA gene family in wheat and related species. Genome 32, 1003–1016. doi: 10.1139/g89-545

[B14] Dyck P. (1992). Transfer of a gene for stem rust resistance from *Triticum araraticum* to hexaploid wheat. Genome 35, 788–792. doi: 10.1139/g92-120

[B15] Faegri K. van der Pijl L. (1979). Abiotic Pollination. Principles of Pollination Ecology (Amsterdam: Pergamon). doi: 10.1016/C2009-0-00736-3

[B16] Garst N. (2017). Assessing anther extrusion and its effect on US hard winter wheat (*Triticum aestivum* L.) hybrid seed production. Thesis at the university of Nebraska, 1–37.

[B17] Garst N. Belamkar V. Easterly A. Guttieri M. J. Stoll H. H. Ibrahim A. M.. (2023). Evaluation of pollination traits important for hybrid wheat development in Great Plains germplasm. Crop Sci. 63, 1169–1182. doi: 10.1002/csc2.20926

[B18] Ge X. Chang F. Ma H. (2010). Signalling and transcriptional control of reproductive development in arabidopsis. Curr. Biol. 20, 988–997. doi: 10.1016/j.cub.2010.09.040, PMID: 21093795

[B19] Goss J. A. (1968). Development, physiology, and biochemistry of corn and wheat pollen. Bot. Rev. 34, 333–359. doi: 10.1007/Bf02985391

[B20] Grewal S. Guwela V. Newell C. Yang C.-Y. Ashling S. Scholefield D.. (2021). Generation of doubled haploid wheat-*Triticum urartu* introgression lines and their characterisation using chromosome-specific KASP markers. Front. Plant Sci. 12. doi: 10.3389/fpls.2021.643636, PMID: 34054892 PMC8155260

[B21] Grewal S. Hubbart-Edwards S. Yang C. Devi U. Baker L. Heath J.. (2019). Rapid Identification of homozygous, and site of wild relative introgressions in wheat through chromosome-specific KASP genotyping assays. Plant Biotechnol. J.l 18, 743–755. doi: 10.1111/pbi.13241, PMID: 31465620 PMC7004896

[B22] Grewal S. Hubbart-Edwards S. Yang C. Scholefield D. Ashling S. Burridge A.. (2018). Detection of *T. urartu* introgressions in wheat and development of a panel of interspecific introgression lines. Front. Plant Sci. 9. doi: 10.3389/fpls.2018.01565, PMID: 30420865 PMC6216105

[B23] Grewal S. Othmeni M. Walker J. Hubbart-Edwards S. Yang C.-Y. Scholefield D.. (2020). Development of Wheat-*Aegilops caudata* introgression lines and their characterisation using genome-specific KASP markers. Front. Plant Sci. 11. doi: 10.3389/fpls.2020.00606, PMID: 32477394 PMC7240103

[B24] Grewal S. Yang C. Scholefield D. Ashling S. Ghosh S. Swarbreck D.. (2024). Chromosome-scale genome assembly of bread wheat’s wild relative *Triticum timopheevii* . Sci. Data 11, 420. doi: 10.1038/s41597-024-03260-w, PMID: 38653999 PMC11039740

[B25] Gupta P. K. Balyan H. S. Gahlaut V. Saripalli G. Pal B. Basnet B. R.. (2019). Hybrid wheat: past, present, and future. Theor. Appl. Genet. 132, 2463–2483. doi: 10.1007/s00122-019-03397-y, PMID: 31321476

[B26] Hegde S. G. Waines J. G. (2004). Hybridization and Introgression between bread wheat and wild and weedy relatives in North America. Crop Sci. 44, 1145–1155. doi: 10.2135/cropsci2004.1145

[B27] Heslop−Harrison Y. J. S. Heslop−Harrison J. S. (1996). Lodicule function and filament extension in the grasses: potassium ion movement and tissue specialization. Ann. Bot. 77, 573–582. doi: 10.1093/aob/77.6.573

[B28] IWGSC Eversole K. Feuillet C. Keller B. Rogers J. Stein N.. (2018). Shifting the limits in wheat research and breeding using a fully annotated reference genome. Science 17, 361. doi: 10.1126/science.aar7191, PMID: 30115783

[B29] Järve K. Peusha H. O. Tsymbalova J. Tamm S. Devos K. M. Enno T. M. (2000). Chromosomal location of a *Triticum timopheevii*-derived powdery mildew resistance gene transferred to common wheat. Genome 43, 377–381. doi: 10.1139/g99-141, PMID: 10791827

[B30] Joppa L. R. McNeal F. H. Berg M. A. (1968). Pollen production and pollen shedding of hard red spring (*Triticum aestivum* L. em. Thell.) and durum (*T. durum* Desf.) wheats. Crop Sci. 8, 487–490. doi: 10.2135/cropsci1968.0011183X000800040028x

[B31] Khare V. Srivastava P. Sharma A. Bains N. S. Sinha K. (2018). Rye confers high anther extrusion to bread wheat via triticale x wheat crosses. J. Pharm. Phytochem. 7, 2167–2170.

[B32] Khazan S. Minz-Dub A. Sela H. Manisterski J. Ben-Yehuda P. Sharon A.. (2020). Reducing the size of an alien segment carrying leaf rust and stripe rust resistance in wheat. BMC Plant Biol. 20, 1–13. doi: 10.1186/s12870-020-2306-9, PMID: 32272895 PMC7147030

[B33] Khlebova L. Barysheva N. V. (2016). Genetic control of resistance to stem rust in durum wheat introgressive lines derived from *Triticum timopheevii* Zhuk. Ukr. J. Ecol. 6, 121–131. doi: 10.15421/201678

[B34] King J. Grewal S. Othmeni M. Coombes B. Yang C. Y. Walter N.. (2022). Introgression of the *Triticum timopheevii* genome into wheat detected by chromosome-specific Kompetitive Allele Specific PCR markers. Front. Plant Sci. 13. doi: 10.3389/fpls.2022.919519, PMID: 35720607 PMC9198554

[B35] King J. Grewal S. Yang C. Hubbart S. Scholefield D. Ashling S.. (2017). A step change in the transfer of interspecific variation into wheat from *Amblyopyrum muticum* . Plant Biotechnol. J. 15, 217–226. doi: 10.1111/pbi.12606, PMID: 27459228 PMC5258861

[B36] King J. Newell C. Grewal S. Hubbart-Edwards S. Yang C. Y. Scholefield D.. (2019). Development of stable homozygous wheat/*Amblyopyrum muticum* (*Aegilops mutica*) introgression lines and their cytogenetic and molecular characterization. Front. Plant Sci. 10. doi: 10.3389/fpls.2019.00034, PMID: 30792722 PMC6365885

[B37] Koebner R. M. D. Shepherd K. W. (1988). “Isolation and agronomic assessment of allosyndetic recombinants derived from wheat/rye translocation 1DL.1RS, carrying reduced amounts of rye chromatin,” in Proceedings of the 7th International Wheat Genetics Symposium. Eds. Miller T. E. Koebner R. M. D. (Institution of Plant Science Research, Cambridge), 343–348.

[B38] Koebner R. M. Shepherd K. W. Appels R. (1986). Controlled introgression to wheat of genes from rye chromosome arm 1RS by induction of allosyndesis: 2. Characterisation of recombinants. Theor. Appl. Genet. 73, 209–217. doi: 10.1007/BF00289276, PMID: 24240852

[B39] Langer S. M. Longin C. F. H. Würschum T. (2014). Phenotypic evaluation of floral and flowering traits with relevance for hybrid breeding in wheat (*Triticum aestivum* L.). Plant Breed. 133, 433–441. doi: 10.1111/pbr.12192

[B40] Lelley J. (1966). Befruchtungsbiologische Beobachtungen im Zusammenhang mit der Saatguterzeugung von Hybridweizen. Zuchter. Genet. Breed. Res. 36, 314–317. doi: 10.1007/BF00446050

[B41] Leonova I. N. Budashkina E. B. Flath K. Weidner A. Börner A. Röder M. S. (2010). Microsatellite mapping of a leaf rust resistance gene transferred to common wheat from *Triticum timopheevii* . Cereal Res. Commun. 38, 211–219. doi: 10.1556/CRC.38.2010.2.7

[B42] Longin C. F. H. Muhleisen J. Maurer H. P. Zhang H. Gowda M. Reif J. C. (2012). Hybrid breeding in autogamous cereals. Theor. Appl. Genet. 125, 1087–1096. doi: 10.1007/s00122-012-1967-7, PMID: 22918662

[B43] Lu Q. Lillemo M. Skinnes H. He X. Shi J. Ji F.. (2013). Anther extrusion and plant height are associated with Type I resistance to Fusarium head blight in bread wheat line ‘Shanghai-3/Catbird’. Theor. Appl. Genet. 126, 317–334. doi: 10.1007/s00122-012-1981-9, PMID: 23052019

[B44] Lukaszewski A. J. Cowger C. (2017). Re-engineering of the *Pm21* transfer from *Haynaldia villosa* to bread wheat by induced homoeologous recombination. Crop Sci. 57, 2590–2594. doi: 10.2135/cropsci2017.03.0192

[B45] Lumivero (2023). XLSTAT statistical and data analysis solution. Available online at: https://www.xlstat.com/en (Accessed July 14, 2025).

[B46] McIntosh R. Gyarfas J. (1971). *Triticum timopheevii* as a source of resistance to wheat stem rust. Z. für Pflanzenzuchtung 66, 240–248.

[B47] Melonek J. Duarte J. Martin J. Beuf L. Murigneux A. Varenne P.. (2021). The genetic basis of cytoplasmic male sterility and fertility restoration in wheat. Nat. Commun. 12, 1036. doi: 10.1038/s41467-021-21225-0, PMID: 33589621 PMC7884431

[B48] Murai K. Takumi S. Koga H. Ogihara Y. (2002). Pistillody, homeotic transformation of stamens into pistil-like structures, caused by nuclear-cytoplasm interaction in wheat. Plant J. 29, 169–181. doi: 10.1046/j.0960-7412.2001.01203.x, PMID: 11851918

[B49] Nguyen V. Fleury D. Timmins A. Laga H. Hayden M. Mather D.. (2015). Addition of rye chromosome 4R to wheat increases anther length and pollen grain number. Theor. Appl. Genet. 128, 953–964. doi: 10.1007/s00122-015-2482-4, PMID: 25716820

[B50] Ogihara Y. Tsunewaki K. (1988). Diversity and evolution of chloroplast DNA in *Triticum* and *Aegilops* as revealed by restriction fragment analysis. Theor. Appl. Genet. 76, 321–332. doi: 10.1007/BF00265331, PMID: 24232195

[B51] Perugini L. D. Murphy J. P. Marshall D. Brown-Guedira G. (2008). *Pm37*, a new broadly effective powdery mildew resistance gene from *Triticum timopheevii* . Theor. Appl. Genet. 116, 417–425. doi: 10.1007/s00122-007-0679-x, PMID: 18092148

[B52] Pickett A. (1993). Hybrid wheat: results and problems. J. Agric. Sci. 121, 294–294. doi: 10.1017/S002185960007725X

[B53] Rodriguez S. Maestra B. Perera E. Diez M. Naranjo T. (2000). Pairing affinities of the B-and G-genome chromosomes of polyploid wheats with those of *Aegilops* sp*eltoides* . Genome 43, 814–819. doi: 10.1139/g00-055, PMID: 11081971

[B54] Sade B. Ibrahim A. M. H. Subramanian N. Rudd J. Liu S. Opena G.. (2022). Assessment of floral characteristics for hybrid wheat (*Triticum aestivum* L.) production in Texas. Agrosystems Geosci. Environ. 5, 1–11. doi: 10.1002/agg2.20228

[B55] Sage G. de Isturiz M. J. (1974). The inheritance of anther extrusion in two spring wheat varieties. Theor. Appl. Genet. 45, 126–133. doi: 10.1007/BF00291142, PMID: 24419327

[B56] Schneider J. Frels K. Sakhale S. Baenziger P. S. Longin C. F. H. Reif J. C.. (2024). Reciprocal Evaluation of Hybrid Wheat (*Triticum aestivum* L.) Crosses Between German and US ‘Great Plains’ genotypes across their contrasting target environments. Plant Breed. 144, 22–34. doi: 10.1111/pbr.13220

[B57] Sears E. R. (1956). The transfer of leaf rust resistance from *Aegilops umbellulata* to wheat. Brookhaven Symp. Biol. 9, 1–21.

[B58] Selva C. Riboni M. Baumann U. Würschum T. Whitford R. Tucker M. R. (2020). Hybrid breeding in wheat: How shaping floral biology can offer new perspectives. Funct.l Plant Biol. 47, 675–694. doi: 10.1071/FP19372, PMID: 32534601

[B59] Singh A. K. Sharma J. B. Vinod Singh P. K. Singh A. Mallick N. (2017). Genetics and mapping of a new leaf rust resistance gene in *Triticum aestivum* L. × *Triticum timopheevii* Zhuk. derivative ‘Selection G12’. J. Genet. 96, 291–297. doi: 10.1007/s12041-017-0760-4, PMID: 28674228

[B60] Singh S. P. Srivastava R. Kumar J. (2015). Male sterility systems in wheat and opportunities for hybrid wheat development. Acta Physiol. Plant 37, 1713. doi: 10.1007/s11738-014-1713-7

[B61] Skinnes H. Semagn K. Tarkegne Y. Maroy A. G. Bjornstad A. (2010). The inheritance of anther extrusion in hexaploid wheat and its relationship to Fusarium head blight resistance and deoxynivalenol content. Plant Breed. 129, 149–155. doi: 10.1111/j.1439-0523.2009.01731.x

[B62] Steed A. King J. Grewal S. Yang C. Y. Clarke M. Devi U.. (2022). Identification of Fusarium head blight resistance in *Triticum timopheevii* accessions and characterization of wheat-*T. timopheevii* introgression lines for enhanced resistance. Front. Plant Sci. 13. doi: 10.3389/fpls.2022.943211, PMID: 35874002 PMC9298666

[B63] Suresh Bishnoi Om P. (2020). A review on hybrid wheat: Problems and prospective. Int. J. Curr. Adv. Res. 8, 18321–18323. doi: 10.24327/ijcar.2019.18323.3500

[B64] Tadlock J. K. (2015). Floral characteristics and hybrid performance of potential candidates of a hybrid wheat (*Triticum aestivum L.*) program in Texas. Texas A&M University, College Station, Texas.

[B65] Ter Steeg E. M. S. Struik P. C. Visser R. G. F. Lindhout P. (2022). Crucial factors for the feasibility of commercial hybrid breeding in food crops. Nat. Plants 8, 463–473. doi: 10.1038/s41477-022-01142-w, PMID: 35513713

[B66] Thomson D. Henry R. (1995). Single-step protocol for preparation of plant tissue for analysis by PCR. Biotechniques 19, 394–397, 400. 7495552

[B67] Uhrin A. Szakács É. Láng L. Bedö Z. Molnár-Láng M. (2012). Molecular cytogenetic characterization and SSR marker analysis of a leaf rust resistant wheat line carrying a 6G(6B) substitution from *Triticum timopheevii* (Zhuk.). Euphytica 186, 45–55. doi: 10.1007/s10681-011-0483-1

[B68] Vogel O. A. (1941). Relation of glume strength and other characters to shattering in wheat. Agron. J. 33, 583–589. doi: 10.2134/agronj1941.00021962003300070001x

[B69] Waines J. G. Hegde S. G. (2003). Intraspecific gene flow in bread wheat as affected by reproductive biology and pollination ecology of wheat flowers. Crop Sci. 43, 451–463. doi: 10.2135/cropsci2003.4510

[B70] Whitford R. Fleury D. Reif J. C. Garcia M. Okada T. Korzun V.. (2013). Hybrid breeding in wheat: technologies to improve hybrid wheat seed production. J. Exp. Bot. 64, 5411–5428. doi: 10.1093/jxb/ert333, PMID: 24179097

[B71] Wilson J. Ross W. M. (1962). Male sterility interaction of the *Triticum aestivum* nucleus and *Triticum timopheevii* cytoplasm. Wheat Inf. Serv. 14, 29–30.

[B72] Zhang G. Mergoum M. Kianian S. Meyer D. W. Simsek S. Singh P. K. (2009). Genetic relationship and QTL association between kernel shattering and agronomic traits in wheat. Crop Sci. 49, 451–458. doi: 10.2135/cropsci2008.06.0329

