# Supplementary material for: Exploring the genetic variation of wheat-Triticum timopheevii introgression lines for flowering morphology traits for hybrid wheat use
Source: Front Plant Sci. 2025 Aug 19;16:1621725. doi: 10.3389/fpls.2025.1621725 (PMC12401957; doi:10.3389/fpls.2025.1621725)
Supplement: Supplementary Figure 1 — KASP marker identification of introgressions present in the 24 wheat-T. timopheevii introgression lines screened for flowering morphology. [file Image1.pdf]

Table 1. KASP marker identification of introgressions present in the 24 wheat-*T.timopheevii* introgression lines screened for flowering morphology.

Legend		
	a	**Homozygouse wheat call**
	b	**Homozygouse T. timopheevii call**

Chromosomes	Marker ID	Position	1At.A1	1At.A5	1At.A7	1At.A9	1At.A12
**1At/1A**	**WRC2170**	064	b	a	a	a	a
	**WRC2172**	508	a	b	a	a	a
	**WRC2173**	658	a	b	a	a	a
	**WRC0010**	1010	a	b	a	a	a
	**WRC2174**	1477	a	b	a	a	a
	**WRC0013**	1802	a	b	a	a	a
	**WRC2175**	2191	a	b	a	a	a
	**WRC2367**	2629	a	b	a	a	a
	**WRC0022**	2917	a	b	a	a	a
	**WRC0024**	3332	a	b	a	a	a
	**WRC0025**	3430	a	b	a	a	a
	**WRC0026**	3657	a	b	a	a	a
	**WRC2177**	4011	a	b	a	a	a
	**WRC0032**	4394	a	b	b	a	a
	**WRC2243**	4738	a	b	b	a	a
	**WRC0040**	5171	a	a	b	b	a
	**WRC0042**	5221	a	a	b	b	a
	**WRC2368**	5627	a	a	a	a	a
	**WRC2180**	5804	a	a	a	a	b

Chromosomes	Marker ID	Position	2At.A1	2At.A5	2At.A10
**2At/2A**	**WRC2186**	013	b	a	a
	**WRC2187**	105	a	a	a
	**WRC0149**	150	a	a	a
	**WRC0151**	165	a	a	a
	**WRC2188**	502	a	b	a
	**WRC0153**	818	a	b	a
	**WRC0154**	1191	a	b	a
	**WRC0156**	1270	a	b	a
	**WRC0161**	1669	a	b	a
	**WRC0163**	1922	a	b	a
	**WRC1329**	2071	a	b	a
	**WRC0164**	2408	a	b	a
	**WRC2190**	3075	a	b	a
	**WRC0169**	3337	a	b	a
	**WRC0172**	3980	a	b	a
	**WRC2192**	4490	a	b	a
	**WRC0178**	4749	a	b	a
	**WRC1433**	5142	a	b	a
	**WRC0180**	5296	a	b	a
	**WRC0181**	5555	a	b	a
	**WRC0183**	5600	a	b	a
	**WRC0188**	6086	a	b	a
	**WRC2193**	6356	a	b	a
	**WRC0191**	6730	a	a	a
	**WRC0193**	7069	a	a	a
	**WRC2194**	7377	a	a	a
	**WRC2196**	7620	a	a	b

Chromosomes

3At/2A

Marker ID	Position	3At.A1
**WRC2203**	013	b
**WRC2246**	120	a
**WRC0295**	275	a
**WRC2247**	338	a
**WRC0302**	837	a
**WRC1616**	1251	a
**WRC0307**	1759	a
**WRC0308**	1759	a
**WRC2205**	2088	a
**WRC0312**	2500	a
**WRC0314**	3120	a
**WRC2248**	3642	a
**WRC0317**	4010	a
**WRC2208**	4463	a
**WRC2209**	4577	a
**WRC2210**	5062	a
**WRC0326**	5504	a
**WRC2211**	5134	a
**WRC0327**	5681	a
**WRC1467**	6087	a
**WRC0332**	6430	a
**WRC2212**	6918	a
**WRC2213**	7193	a
**WRC2214**	7419	a

Chromosomes	Marker ID	Position	5At.A1	5At.A8
**5At/5A**	**WRC2272**	064	b	a
	**WRC0560**	134	a	a
	**WRC2273**	410	a	a
	**WRC1646**	466	a	a
	**WRC0566**	911	a	a
	**WRC0569**	1136	a	a
	**WRC2275**	1745	a	a
	**WRC0577**	1947	a	a
	**WRC2276**	2166	a	a
	**WRC0579**	2679	a	a
	**WRC2277**	3005	a	a
	**WRC0581**	3274	a	a
	**WRC1519**	3580	a	a
	**WRC2278**	3781	a	a
	**WRC0586**	4270	a	a
	**WRC2279**	4729	a	b
	**WRC1522**	5014	a	b
	**WRC2280**	5184	a	b
	**WRC0597**	5498	a	b
	**WRC1135**	5757	a	b
	**WRC1136**	5757	a	b
	**WRC0599**	5955	a	b
	**WRC2281**	6247	a	a
	**WRC2094**	6378	a	a
	**WRC2282**	6546	a	a
	**WRC1138**	6771	a	a
	**WRC0605**	6887	a	a
	**WRC0606**	7020	a	a
	**WRC2283**	7084	a	a
	**WRC1526**	7088	a	a

5At.A10	5At.A13	5At.A17	5At.A24
a	a	a	a
a	a	a	a
a	a	a	a
a	a	a	a
a	a	a	a
a	a	a	a
a	a	a	a
a	a	a	a
a	a	a	a
a	a	a	a
a	a	a	a
a	a	a	a
a	a	a	a
a	a	a	a
a	a	a	a
a	a	a	a
b	a	a	a
b	a	a	a
b	b	a	a
a	b	b	a
a	b	b	a
a	a	b	a
a	a	b	a
a	a	b	a
a	a	b	a
a	a	b	a
a	a	a	a
a	a	a	a
a	a	a	b
a	a	a	b

Chromosomes	Marker ID	Position	6At.A3
6At/6A	**WRC0720**	577	a
	**WRC2295**	934	a
	**WRC2296**	1351	a
	**WRC2297**	1734	a
	**WRC0730**	2227	a
	**WRC0731**	2351	a
	**WRC2298**	3059	b
	**WRC1019**	3579	a
	**WRC2300**	3949	a
	**WRC0738**	4323	a
	**WRC2301**	4835	a
	**WRC2302**	4960	a
	**WRC0747**	5350	a
	**WRC0750**	5876	a
	**WRC1206**	5956	a
	**WRC2100**	6000	a
	**WRC0754**	6044	a
	**WRC0755**	6049	a
	**WRC2303**	6119	a
	**WRC2304**	6149	a

6At

6At.A7	6At.A9
a	a
a	a
a	a
a	a
a	a
a	a
a	a
a	a
a	a
a	a
a	a
a	a
a	a
b	a
b	b
a	a
a	a
a	a
a	a
a	a

Chromosomes	Marker ID	Position	7At.A1	7At.A5	7At.A8
7At/7A	**WRC1861**	036	b	b	a
	**WRC0866**	421	a	b	a
	**WRC0868**	837	a	b	a
	**WRC0869**	1289	a	b	b
	**WRC1380**	1337	a	b	b
	**WRC0871**	1729	a	b	b
	**WRC2317**	2012	a	b	b
	**WRC0879**	2470	a	a	b
	**WRC2395**	2996	a	a	b
	**WRC2396**	3030	a	a	b
	**WRC0884**	3324	a	a	b
	**WRC2318**	3963	a	a	b
	**WRC2321**	5150	a	a	b
	**WRC1382**	5419	a	a	a
	**WRC0892**	5533	a	a	a
	**WRC0893**	5564	a	a	a
	**WRC2322**	6030	a	a	a
	**WRC0895**	6354	a	a	a
	**WRC1026**	7178	a	a	a
	**WRC2324**	7321	a	a	a
	**WRC2325**	7337	a	a	a
	**WRC1577**	7366	a	a	a

7At.A9	7At.A12	Chromosomes	Marker ID	Position	1G.B1
a	a	1G/1B	**WRC2183**	070	b
a	a		**WRC2369**	287	a
a	a		**WRC1404**	508	a
b	a		**WRC0052**	667	a
b	a		**WRC0055**	962	a
b	a		**WRC0060**	1304	a
b	a		**WRC2374**	1331	a
b	a		**WRC2244**	1780	a
b	a		**WRC0066**	2181	a
b	a		**WRC1408**	2711	a
b	a		**WRC1084**	2820	a
b	a		**WRC0073**	3265	a
b	a		**WRC0076**	3332	a
b	b		**WRC2034**	3657	a
b	b		**WRC0079**	4091	a
b	b		**WRC2035**	4236	a
b	b		**WRC1054**	4415	a
b	b		**WRC0083**	4664	a
a	a		**WRC0085**	5104	a
a	a		**WRC0091**	5680	a
a	a		**WRC0095**	6035	a
a	a		**WRC1067**	6287	a
			**WRC0096**	6289	a
			**WRC0098**	6696	a
			**WRC0099**	6696	a
			**WRC0100**	6696	a
			**WRC1598**	6887	a

Chromosomes	Marker ID	Position	2G.B12	2G.B14	2G.B15	Chromosomes
**2G/2B**	**WRC0197**	074	a	a	a	**2G/2D**
	**WRC0198**	074	a	a	a	
	**WRC0199**	074	a	a	a	
	**WRC2199**	249	a	a	a	
	**WRC2200**	433	a	a	a	
	**WRC0202**	1055	a	a	a	
	**WRC0203**	1223	a	a	a	
	**WRC1441**	1501	a	a	a	
	**WRC0205**	1655	b	a	a	
	**WRC0208**	1887	b	a	a	
	**WRC1336**	2091	b	a	a	
	**WRC0210**	2450	b	a	a	
	**WRC0211**	2587	b	a	a	
	**WRC1444**	3135	b	a	a	
	**WRC2230**	1933	b	a	a	
	**WRC0215**	3354	b	a	a	
	**WRC0219**	3606	b	a	a	
	**WRC0222**	4217	b	a	a	
	**WRC0225**	4457	b	b	a	
	**WRC0226**	4540	b	b	a	
	**WRC0228**	4966	b	b	a	
	**WRC0229**	5393	b	b	a	
	**WRC0230**	5393	b	b	a	
	**WRC0232**	5926	b	b	a	
	**WRC1448**	6030	b	b	a	
	**WRC0235**	6255	b	b	a	
	**WRC0236**	6255	b	b	a	
	**WRC0238**	6361	b	b	a	
	**WRC0239**	6361	b	b	a	
	**WRC2048**	6732	b	b	a	
	**WRC0242**	7110	b	b	a	
	**WRC2051**	7286	b	b	a	
	**WRC1098**	7340	b	b	a	
	**WRC0244**	7433	b	b	a	
	**WRC0249**	7788	b	a	b	

Marker ID	Position	2G.D1	2G.D2	2G.D4	2G.D5
**WRC2059**	026	b	b	a	a
**WRC0255**	367	b	b	a	a
**WRC2061**	1730	b	b	b	a
**WRC2377**	1998	b	b	b	b
**WRC0268**	3004	b	b	b	b
**WRC0276**	4104	b	b	b	b
**WRC0277**	4229	b	b	b	b
**WRC0280**	4805	a	b	b	b
**WRC0289**	6115	a	a	a	a
**WRC0292**	6272	a	a	a	a
**WRC2202**	6488	a	a	a	a

Chromosomes	Marker ID
**3G/3B**	**WRC2217**
	**WRC2068**
	**WRC0339**
	**WRC0340**
	**WRC0341**
	**WRC2218**
	**WRC2219**
	**WRC2220**
	**WRC2221**
	**WRC2222**
	**WRC2223**
	**WRC2225**
	**WRC2226**
	**WRC2227**
	**WRC2228**
	**WRC0348**
	**WRC0350**
	**WRC0352**
	**WRC0353**
	**WRC2229**
	**WRC2231**
	**WRC0361**
	**WRC2232**
	**WRC2233**
	**WRC2234**
	**WRC0366**
	**WRC0367**
	**WRC2235**
	**WRC1473**
	**WRC1117**
	**WRC1348**
	**WRC0376**
	**WRC0379**
	**WRC0381**
	**WRC0382**
	**WRC2379**

WRC0384

WRC2236

WRC0389

WRC0390

WRC2237

WRC2238

Position	3G.B1	3G.B2	3G.B4	3G.B5
045	b	b	b	b
108	a	b	b	b
129	a	a	b	b
129	a	a	b	b
129	a	a	b	b
202	a	a	b	b
218	a	a	b	b
271	a	a	b	b
280	a	a	b	b
305	a	a	b	b
398	a	a	b	b
435	a	a	b	b
488	a	a	b	b
674	a	a	a	b
674	a	a	a	b
771	a	a	a	b
771	a	a	a	b
1272	a	a	a	b
1324	a	a	a	b
1802	a	a	a	b
2284	a	a	a	b
2513	a	a	a	b
2997	a	a	a	b
3369	a	a	a	b
3592	a	a	a	b
3858	a	a	a	b
4051	a	a	a	b
4486	a	a	a	b
4519	a	a	a	b
4989	a	a	a	b
5760	a	a	a	b
5951	a	a	a	b
6208	a	a	a	b
6690	a	a	a	b
6724	a	a	a	b
7155	a	a	a	b

Chromosomes	Marker ID	Position
4G/4B	WRC2264	013
	WRC0482	050
	WRC0484	222
	WRC1363	285
	WRC0485	285
	WRC2380	522
	WRC2265	599
	WRC2381	799
	WRC0489	1030
	WRC0490	1699
	WRC0494	2071
	WRC0495	2476
	WRC0496	2677
	WRC1502	3067
	WRC2266	3163
	WRC0498	3516
	WRC0500	3738
	WRC0503	4142
	WRC2382	4633
	WRC0508	5202
	WRC0511	5629
	WRC2269	6141
	WRC0514	6495
	WRC1367	6573

7368	a	a	a	b
7622	a	a	a	b
8072	a	a	a	a
8072	a	a	a	a
8166	a	a	a	a
8292	a	a	a	a

4G.B1	4G.B2
b	a
a	a
a	a
a	a
a	a
a	a
a	a
a	a
a	a
a	a
a	a
a	a
a	a
a	a
a	a
a	a
a	a
a	a
a	a
a	b
a	b
a	b
a	b
a	b

Chromosomes	Marker ID	Position	5G.B3	5G.B8	5G.B14
5G/5B	**WRC2284**	051	b	a	a
	**WRC0609**	104	b	a	a
	**WRC0610**	104	b	a	a
	**WRC1143**	176	b	a	a
	**WRC0612**	176	b	a	a
	**WRC0613**	413	b	a	a
	**WRC0615**	413	b	a	a
	**WRC2095**	825	b	a	a
	**WRC2096**	941	b	a	a
	**WRC2285**	1283	b	a	a
	**WRC0619**	922	b	a	a
	**WRC0623**	1581	b	a	a
	**WRC2286**	2013	b	a	a
	**WRC0625**	2473	b	a	a
	**WRC1530**	2543	b	a	a
	**WRC2384**	2996	b	a	a
	**WRC2387**	3829	b	a	a
	**WRC2388**	3974	b	a	a
	**WRC0640**	4414	b	a	a
	**WRC0644**	4857	b	a	a
	**WRC0646**	4961	b	a	a
	**WRC2289**	5343	b	b	a
	**WRC0650**	5607	b	b	a
	**WRC0651**	5801	b	b	a
	**WRC2290**	6103	a	b	a
	**WRC1654**	6110	a	b	a
	**WRC0657**	6584	a	b	b
	**WRC0658**	6731	a	b	b
	**WRC2391**	7050	a	b	b

5G.B15
a
a
a
a
a
a
a
a
a
a
a
a
a
a
a
a
a
a
a
a
a
a
a
a
a
a
a
a
b

Chromosomes	Marker ID	Position	6G.B3	6G.B4	6G.B5
6G/6B	WRC2305	127	a	a	a
	WRC0760	362	a	a	a
	WRC1167	468	b	a	a
	WRC0762	501	b	a	a
	WRC0763	618	b	a	a
	WRC0764	902	b	a	a
	WRC2306	1498	b	a	a
	WRC2307	1811	b	a	a
	WRC1211	1857	b	a	a
	WRC0773	2218	b	a	a
	WRC0775	2588	b	a	a
	WRC0776	2588	b	a	a
	WRC2394	3081	b	a	a
	WRC0780	3394	b	a	a
	WRC0784	3871	b	a	a
	WRC0785	4172	b	a	a
	WRC0786	4223	b	a	a
	WRC2309	4840	b	a	a
	WRC0790	5067	b	a	a
	WRC0794	5419	b	a	a
	WRC2310	5948	b	a	a
	WRC0800	6375	b	a	a
	WRC0802	6414	b	a	a
	WRC0803	6712	a	a	a
	WRC0804	6836	a	a	a
	WRC2312	7143	a	b	a
	WRC1875	7157	a	b	b

Chromosomes	Marker ID	Position	7G.B1	7G.B3	7G.B4	Chromosomes
**7G/7B**	**WRC2326**	035	b	a	a	**7G/7D**
	**WRC2327**	177	b	a	a	
	**WRC1682**	533	b	a	a	
	**WRC0907**	698	b	a	a	
	**WRC1580**	1023	b	a	a	
	**WRC0910**	1154	b	a	a	
	**WRC0914**	1528	b	a	a	
	**WRC2400**	2031	b	a	a	
	**WRC1385**	2556	b	a	a	
	**WRC2329**	3174	b	a	a	
	**WRC1582**	3317	b	a	a	
	**WRC0923**	3876	b	a	a	
	**WRC2401**	4177	b	a	a	
	**WRC0927**	4784	b	a	a	
	**WRC0930**	4798	b	a	a	
	**WRC0931**	4810	b	a	a	
	**WRC2332**	5362	b	a	a	
	**WRC2333**	5823	b	a	a	
	**WRC2334**	6104	b	a	a	
	**WRC0940**	6530	b	a	a	
	**WRC0941**	6931	b	a	a	
	**WRC1392**	6932	b	a	a	
	**WRC2336**	7239	a	a	a	
	**WRC1393**	7394	a	a	a	
	**WRC2337**	7432	a	b	a	
	**WRC2338**	7501	a	b	b	

Marker ID	Position	7G.D2
**WRC1899**	044	b
**WRC0955**	258	b
**WRC0956**	699	b
**WRC0957**	805	b
**WRC0962**	1481	b
**WRC0963**	1575	b
**WRC1713**	2905	b
**WRC0981**	4207	b
**WRC0986**	4612	a
**WRC2339**	4818	a
**WRC0990**	5357	a
**WRC0995**	5932	a
**WRC2340**	6159	a
**WRC2341**	6219	a
