# Supplementary material for: Exploring the genetic variation of wheat-Triticum timopheevii introgression lines for flowering morphology traits for hybrid wheat use
Source: Front Plant Sci. 2025 Aug 19;16:1621725. doi: 10.3389/fpls.2025.1621725 (PMC12401957; doi:10.3389/fpls.2025.1621725)
Supplement: Supplementary Figure 2 — Percentage pollen viability for the wheat parents and controls and the wheat-T. timopheevii introgression lines. [file Image2.pdf]

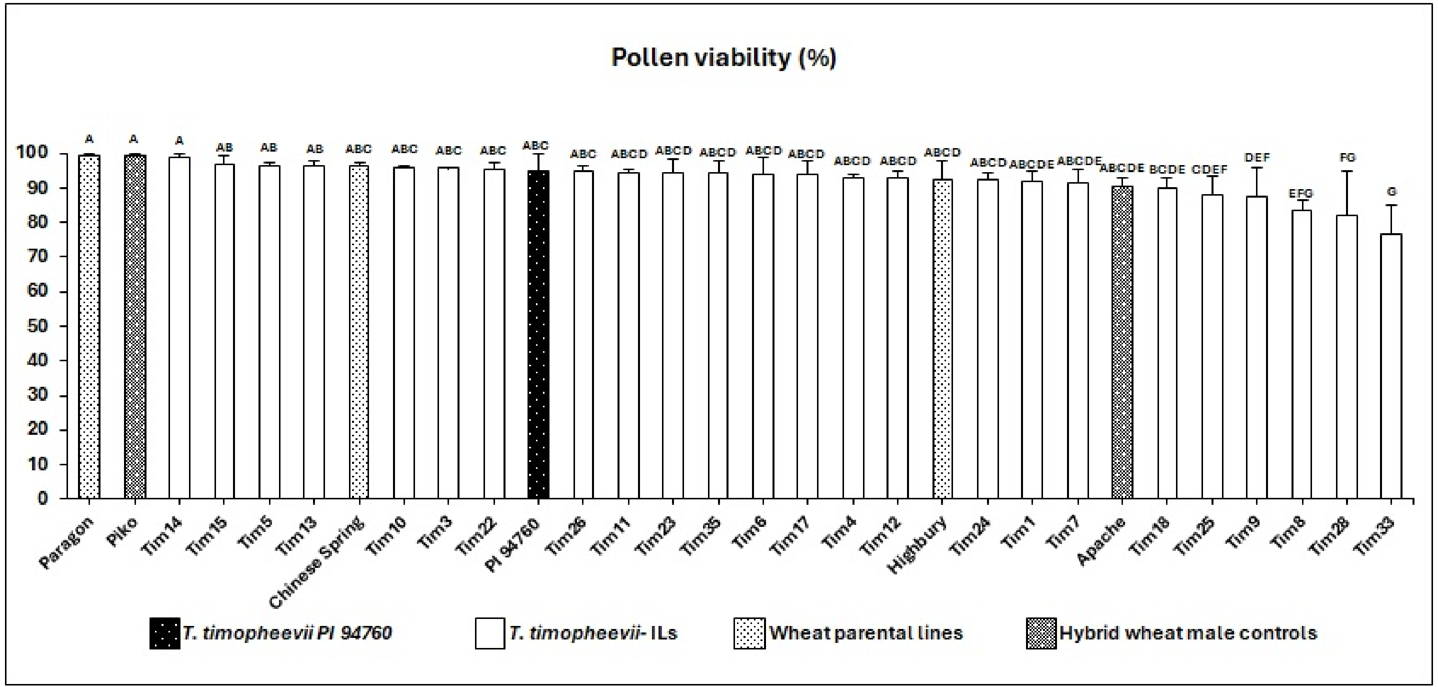

Supplementary Figure 2. Pollen viability data for all wheat-*T. timopheevii* introgression lines. Letters above the bars indicate groupings based on statistical significance. Bars that share the same letter are not significantly different from each other. Bars with different letters are significantly different at p,0.05.
